# Quercetin and Its Structural Analogs as NUDT5 Inhibitors: A Preliminary In Silico Study

**DOI:** 10.3390/ijms26188843

**Published:** 2025-09-11

**Authors:** Emilia Gligorić, Milica Vidić, Branislava Teofilović, Nevena Grujić-Letić

**Affiliations:** Department of Pharmacy, Faculty of Medicine, University of Novi Sad, Hajduk Veljkova 3, 21000 Novi Sad, Serbia; branislava.teofilovic@mf.uns.ac.rs (B.T.); nevena.grujic-letic@mf.uns.ac.rs (N.G.-L.)

**Keywords:** NUDT5, quercetin, structural analog, molecular docking, ADMET

## Abstract

Nucleotide diphosphate hydrolase type 5 (NUDT5) plays a significant role in the estrogen-signaling pathway and is overexpressed in breast cancer. This study aimed to explore the anti-breast cancer potential of quercetin and its 52 structural analogs by targeting the NUDT5 enzyme using the in silico molecular docking method. Moreover, Molecular Mechanics/General Born Surface Area (MM/GBSA) calculations were performed for compounds with superior binding affinity scores than quercetin. Their drug-likeness, according to Lipinski’s rule of five, water solubility, and Caco-2 permeability were predicted. In addition, the absorption, distribution, metabolism, excretion, and toxicity (ADMET) profile was determined for the top-scoring compounds from the docking studies and MM/GBSA calculations, as well as for those that complied with the rules of Lipinski and exhibited high permeability. The obtained results showed that all the tested ligands interact with the active site of NUDT5. Their binding energies ranged from −11.24 to −7.36 kcal/mol. The MM/GBSA calculations further supported the binding affinity predictions. ADMET analysis enabled the selection of compounds with favorable pharmacokinetic profiles in comparison to quercetin. Quercetin analogs L1 and L28 were identified as promising anti-breast cancer drug candidates worthy of further experimental evaluation.

## 1. Introduction

Breast cancer (BRCA) represents a significant health issue for women due to its high mortality and morbidity rates. Even with adjuvant chemotherapy, the five-year survival rate for metastatic BRCA remains below 30% [1]. BRCA is a disease characterized by abnormal and uncontrolled growth of tumor cells in the epithelial tissue of the breast [2]. It is a diverse condition, exhibiting differences in morphology, molecular biology, clinical presentation, and treatment response. Current cancer treatments, including surgery, radiation, and chemotherapy, not only affect cancer cells but healthy cells as well, often leading to toxicity in patients [3,4,5]. Despite advancements in BRCA therapy, resistance to existing treatments remains a significant challenge, highlighting the need for the development of novel anti-BRCA agents that are safer and more effective.

Computational methods have gained significant importance in the design and discovery of new anti-cancer drugs. Computer-aided drug design (CADD) involves the use of computational tools and techniques to identify potential drug targets, screen large molecular databases for potential drug candidates, model and predict interactions between drug molecules and biological targets, and to assess in silico their pharmacokinetic properties and toxicity. The application of computational methods has led to the acceleration of drug discovery by reducing the time and resources needed for experimental evaluation [6,7]. Computational methods have been successfully applied in the development of drugs used to treat various diseases, including cancer [6]. As a result of applying the in silico approach, drugs approved by the Food and Drug Administration (FDA) for the treatment of BRCA were discovered: Alpelisib, Abemaciclib, Talazoparib Tosylate, Neratinib Maleate, and Ribociclib [8].

The application of computational tools is currently the first approach in the discovery and development of new therapeutic agents. In silico studies provide insights into binding modes and interactions between compounds and biological targets at the atomic level, revealing new pathways or targets for cancer therapy, while estimating binding affinities and the potential efficacy of compounds before experimental evaluation. Understanding the molecular mechanisms underlying BRCA and how a specific receptor contributes to tumorigenesis is a crucial aspect of cancer biology that paves the way toward developing effective anti-cancer drugs and improving treatment outcomes.

Nucleotide diphosphate hydrolase type 5 (NUDT5) has been linked to the development of BRCA. Research findings revealed that the elevation of nuclear adenosine triphosphate (ATP), chromatin remodeling, and alterations in gene transcription induced by progestin or estrogen in BRCA cells rely on the activity of NUDT5. Its overexpression is associated with a worse prognosis and a higher risk of recurrence and metastasis [9]. The inhibition of NUDT5 enzymatic activities hinders the formation of oncospheres and prevents the activation of cancer-driver genes. These findings indicate that NUDT5 can be a prognostic biomarker and a target for the discovery of new therapeutic agents in the treatment of estrogen-positive BRCA [10]. Moreover, a study by Qian et al. (2024) highlighted the critical role of NUDT5 in regulating the growth of triple-negative BRCA cells and the potential therapeutic implications of targeting NUDT5 in the treatment of this aggressive type of BRCA [11].

The heterogeneity of BRCA represents a major obstacle in diagnosing this disease and determining adequate therapy for the patient [12]. Estrogen receptor (ER), human epidermal growth factor receptor 2 (HER-2), vascular endothelial growth factor (VEGF), breast cancer gene 1, and breast cancer gene 2 were identified as the main targets for the development of new drugs for the treatment of BRCA. However, there is still a gap in understanding their interaction and interdependence, as well as disease pathogenesis [13]. The inclusion of NUDT5, a protein that affects the proliferation and apoptosis of tumor cells, may be significant for the resolution of some of these concerns. The study of active site residues/binding pockets of NUDT5 protein, as well as NUDT5 inhibitors, using molecular docking and the Molecular Mechanics/Generalized Born Surface Area (MM/GBSA) method would enable the understanding of the binding mode and the development of new drugs in the treatment of BRCA.

NUDT5 plays a crucial role in the estrogen signaling pathway, suggesting that NUDT5 inhibitors can block this pathway and may therefore exhibit similar therapeutic indications as estrogen antagonists. Quercetin has been identified as one of the inhibitors of the estrogen signaling pathway [14], thereby positioning it as a potential NUDT5-targeting compound.

Quercetin (3,3′,4′,5,7-pentahydroxy-2-phenylchromen-4-one) is classified as a flavonol, one of the six subclasses of flavonoid compounds [15]. It is a component of the human diet, present mainly in its glycoside form in a variety of plants, fruits, and vegetables such as onions (*Allium cepa* L.), asparagus (*Asparagus officinalis* L.), and red leaf lettuce (*Lactuca sativa* L.) [16]. Quercetin is also available as a dietary supplement and as a part of functional foods. A growing body of research indicates that quercetin possesses significant therapeutic potential for both preventing and treating various chronic diseases, including cancer. The anti-tumor effects of quercetin have been demonstrated in both in vivo and in vitro experiments [17]. Moreover, quercetin exerts diverse biological effects, such as anti-inflammatory, antioxidant, and neuroprotective. These activities can only be exhibited after its absorption in the human body. However, the absorption of quercetin in its native form is limited, as reflected by the difference between the administered dose and the concentrations detected in plasma [18]. Quercetin is a hydrophobic molecule with relatively low solubility in water, as well as in gastric and small intestinal fluids, which leads to its precipitation in these environments [19]. Nevertheless, quercetin glycosides exhibit high solubility in water, which explains quercetin’s greater bioavailability when consumed as part of dietary sources compared to its aglycone form. Once ingested, quercetin glycosides undergo hydrolysis, releasing the aglycone, which is then absorbed [16]. This process is necessary due to the high polarity of the glycosides, which hinders their passive diffusion and absorption through the phospholipid bilayer of the epithelial cell membrane. Moreover, the absorption of glycosides can occur via active transport in the presence of the sodium-dependent glucose transporter 1 (SGLT1) [19]. After the absorption, quercetin reaches the liver via the portal vein, where it undergoes glucuronidation, methylation, and sulfation before being released into the systemic circulation [20].

The poor water solubility, chemical instability, and short elimination half-life of quercetin limit its application in clinical trials [21]. To overcome these limitations, numerous quercetin derivatives using various methods, including glycosylation, methylation, hydroxylation, and sulfation, have been designed and synthesized [22,23].

Given the urgent need for new therapeutic options in BRCA treatment, it is essential to develop adjuvant agents and drugs with reduced toxicity, fewer adverse effects, higher efficacy, selectivity, and lower risk of resistance. Molecular docking combined with MM/GBSA calculations enables the identification of NUDT5 inhibitors, provides atomic-level insights into NUDT5–ligand interactions, highlights key amino acid residues in the active site, and estimates the stability of protein–ligand complexes. Combined with pharmacokinetic profiling to reduce clinical failure risk, these in silico methods offer a rapid, cost-effective approach to select promising candidates for further in vitro and in vivo studies with an increased probability of success. In this context, the present work aimed to investigate the NUDT5 inhibitory potential of quercetin and its 52 structural analogs using the in silico method of molecular docking and MM/GBSA calculations, as well as predict the absorption, distribution, metabolism, excretion, and toxicity (ADMET) profile of selected quercetin analogs to propose effective orally active anti-BRCA drug candidates.

## 2. Results

### 2.1. Molecular Docking

**Figure 1:**
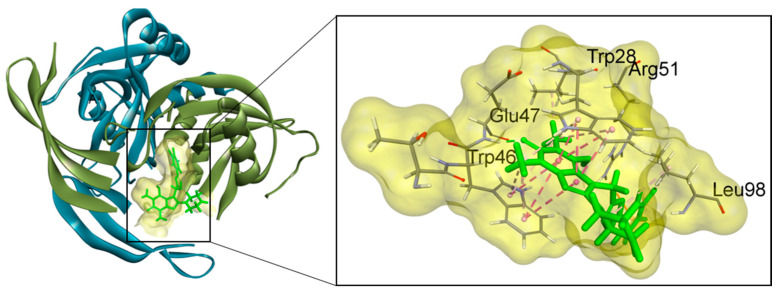
NUDT5-co-crystallized ligand complex (PDB code: 5NWH) with the substrate-binding pocket. The structural and functional core of NUDT5 is composed of four critical amino acid residues, TrpA 28, ArgA 51, TrpB 46 and GluB 47, which are pivotal for ligand binding.

Molecular docking analysis was conducted in order to investigate the anti-BRCA potential of quercetin and its 52 structural analogs (Table S1 in the Supplementary File) by targeting the NUDT5 enzyme. NUDT5 is a homodimer consisting of chain A and chain B. The structure of NUDT5 in complex with its inhibitor (C) (PDB code: 5NWH) [24] gives insight into the substrate-binding cavity as depicted in Figure 1. The key structural and functional features of NUDT5 are the four amino acid residues: Trp28 and Arg51 of chain A and Trp46 and Glu47 of chain B [24].

The conformation characterized by the lowest binding energy for each ligand was regarded as the most favorable. The results of molecular docking analysis for quercetin (L35) and structural analogs (L1–L34) with lower binding energies than quercetin (<−8.0 kcal/mol) are summarized in Table 1. The co-crystallized ligand (C) and two FDA-approved estrogen receptor-positive BRCA drugs—tamoxifen (T) and raloxifene (R)—were used as positive controls. The relevance of T and R as controls has been supported by a previous study by Tong et al. (2020), in which they were used during the evaluation of the cytotoxic effects of NUDT5 inhibitors on BRCA MCF7 cells [14]. The results for ligands (L36–L52) with higher binding energies than quercetin (>−8.0 kcal/mol) are presented in Table S2 in the Supplementary File.

The binding energy values of the analyzed ligands ranged from −11.24 to −7.36 kcal/mol. The lowest binding energy reflects the strongest ligand-macromolecule binding. Data presented in Table 1 indicate that the analyzed compounds are stabilized at the receptor active site by various interactions. It can be observed that the most common amino acid residues that participate in the formation of hydrogen bond are GluB:47, ArgA:51, GlyB:135, and ThrB:45. Also, the most common amino acid residues that participate in establishing van der Waals interactions are as follows: ValA:29, LeuB:136, ArgA:84, ArgB:44, GluA:166, GluA:97, and GluB:135.

### 2.2. Hydrogen Bond Analysis

Due to the essential importance of determining the strength, robustness, and stability of the interactions between the ligands and the target protein, a hydrogen bond analysis was performed. A basic understanding of hydrogen bond strength is made possible by knowing its length and bond angle. Data on the amino acid residues that participate in the construction of the hydrogen bond and the type, length, and angle of the hydrogen bond during the formation of the complex are given in Table 2 for the first five ligands with the lowest value of binding energy, as well as ligands with a favorable pharmacokinetic profile, L35, and controls.

### 2.3. MM/GBSA Binding Free Energy Calculation

The MM/GBSA approach for the evaluation of the binding free energy of ligand molecules was applied as a rescoring parameter to further optimize the lead molecules from the hits obtained from the docking studies. The binding free energies determined for the 35 complexes, as well as the reference compounds, are summarized in Table 3. The binding free energies were decomposed into their energy components: electrostatic, van der Waals energy, SASA non-polar solvation energy, and polar solvation energy to achieve insights into their individual contributions.

It can be observed that among the studied compounds, L5 displayed the highest binding free energy of −47.83 kcal/mol, followed by L10 (−46.94 kcal/mol). These two compounds demonstrated higher binding free energy in comparison to all three reference compounds (C, T, and R). Ligands L1, L2, L3, L8, L9, L14, and L15 displayed higher binding free energy than the reference drugs T and R. The binding free energy of L4 is equal to that of T but lower than that of R. The mentioned ten ligands, along with ligands L17 and L24, possessed lower binding energy than quercetin. From the MM/GBSA calculations, it can be noted that van der Waals energies contribute the most to the binding free energy of the ligands. The electrostatic energy does not contribute either positively or negatively to the binding free energy. Also, the polar solvation energy did not have a favorable contribution to the total interaction in the docked complexes, while the SASA non-polar solvation energy contributed similarly to the binding free energy, as expected from the compounds of similar size.

### 2.4. Lipinski’s Rule of Five and ADMET Profile

Ligands (L1–L34) with lower binding energy values than that of quercetin (L35), as well as L35 and C, were predicted for drug-likeness according to Lipinski’s rules, and a description of their properties, whether they meet or do not meet the rules, is shown in Table 4. In addition, the predicted values for water solubility [log mol/L] and Caco-2 permeability [log cm/s] are also shown. In the SwissADME LogSw scale, compounds with values below −6 are poorly soluble [26]. Solubility of the evaluated ligands is satisfactory. Caco-2 permeability values above 0.9 indicate high permeability and oral activity of a compound [27]. Ligands L17, L24, L28, L30, and L33 showed high permeability.

According to Lipinski’s rules, a molecule is similar to a drug if it has a molecular mass (Mr) ≤ 500 Da, partition coefficient (logP) ≤ 5, number of hydrogen bond acceptors (nHBA) ≤ 10, and number of hydrogen bond donors (nHBD) ≤ 5 [28]. Results in Table 4 showed that eleven ligands out of thirty-six (including C) violate more than one of Lipinski’s rules. Ligands L1, L2, L5, and L10 have two violations with Mr greater than 500 Da and nHBA greater than 10. Ligand L13 also violates two rules with 11 HBA and 7 HBD. Six ligands (L3, L7, L11, L19–L21) do not conform with three criteria of Lipinski’s rules, with Mr greater than 500 Da, nHBA ranging from 14 to 16, and nHBD ranging from 7 to 10. These ligands are less likely to be orally bioavailable. The other molecules tested were within the range of Lipinski’s rules, suggesting that these compounds are absorbed well from the gastrointestinal tract after oral administration.

Furthermore, the pharmacokinetic profile of the top-ranking ligands from the MM/GBSA calculations and of those that comply with Lipinski’s rule and are highly Caco-2 permeable (L17, L24, L28, L30, L33), along with L35, C, and the reference drugs, were fully predicted using the pkCSM web tool. Their ADMET profile is given in Table S3 in the Supplementary File. The intestinal absorption of the selected ligands ranged from 50.2 (L3) to 100% (L15). Ligands L3, L8, and L14 exhibited lower intestinal absorption than quercetin. The volume of distribution (log VDss) ranged from −1.63 (L2) to 1.559 (L35) log L/kg. Ligands displaying log VDss < −0.15 tend to remain in the plasma, while those having log VDss > 0.45 pass into extravascular compartments. Based on the values for blood–brain barrier permeability (log BB < −1), the ligands (except for L28, L30, and L33) do not traverse the blood–brain barrier and do not exert toxic effects on the brain. For metabolism parameters, the results suggested that none of the ligands are substrates or inhibitors of CYP2D6. Furthermore, ligands L3, L8, L14, L28, L30, and L35 are neither substrates nor inhibitors of CYP3A4. In contrast, ligands L2, L4, L5, L9, L10, L15, L17, and L24 are both substrates and inhibitors of CYP3A4, while L1, L10, and L33 are only substrates for CYP3A4. The results also showed that none of the compounds were hepatotoxic or toxic in the AMES test. Although most of the ligands displayed low maximum tolerated dose values (<0.477 log mg/kg/day), they are higher than the values of the reference drugs. For most ligands, the oral rat acute toxicity doses are comparable to the values of the reference drugs. For the majority of ligands, the oral rat chronic toxicity doses are higher than those of the reference drugs.

### 2.5. Toxicity Profile

A more detailed toxicity profile of the top-ranking ligands from the molecular docking studies and MM/GBSA calculations, as well as of highly Caco-2 permeable compounds that comply with Lipinski’s, rule was assessed. The results obtained by ProTox 3.0. Web server are presented in Table 5.

The results indicated that except for L2, L5, L10, and L35, the other ligands showed high LD_50_ values ranging from 1000 to 5000 mg/kg and were categorized into toxicity class 4 or 5. Class 1 indicates high toxicity, whereas class 6 implicates the non-toxic nature of a compound. Ligand L24 displayed the highest LD_50_ value, indicating its low toxic nature. The analyzed ligands demonstrated activity in nephrotoxicity, cardiotoxicity (except for L24, L28, L30, L33, and L35), and immunotoxicity (except for L17, L24, L28, L30, L33, and L35), which indicates potential adverse effects on the kidneys, heart, and immune system. By contrast, the ligands displayed inactivity in hepatotoxicity, neurotoxicity, cytotoxicity, mutagenicity (except for L35), and carcinogenicity (except for L35).

The results of ligand interactions with various receptors of the nuclear signaling pathway, interactions with proteins related to stress response pathways, and molecular initiating events are presented in Tables S4–S6 in the Supplementary File. It is evident that ligands L1, L5, and L8 interact with the smallest number of receptors (1/26), indicating their high selectivity.

### 2.6. Binding Mode Interactions

**Figure 2:**
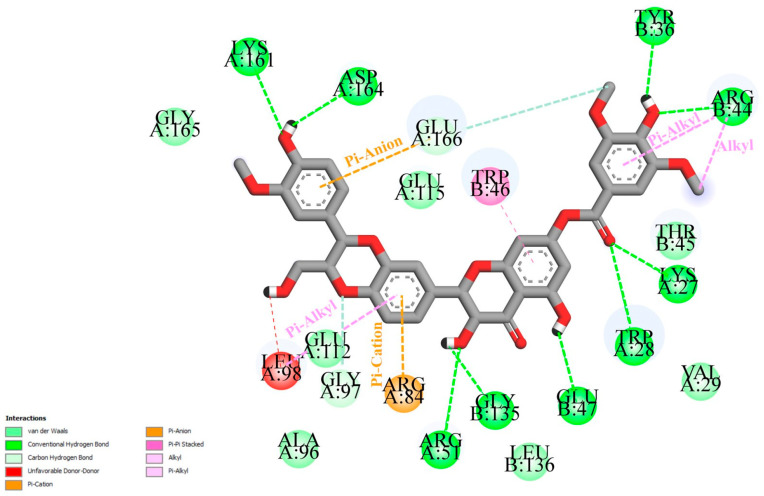
Two-dimensional (2D) representation of the ligand–receptor interactions of L1 at the active site of NUDT5. Amino acid residues involved in ligand interactions are highlighted, with interaction types shown in different colors.

The binding mode interactions of quercetin (L35) and selected structural analogs were explored in more detail. The two-dimensional representation of the interactions of L1 at the receptor site is given in Figure 2.

**Figure 3:**
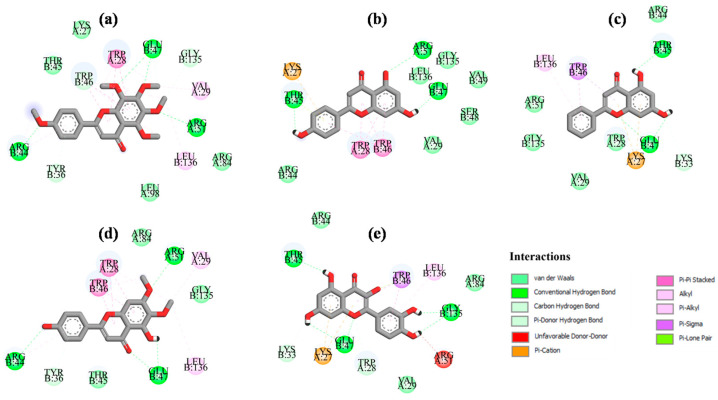
Two-dimensional (2D) representation of the ligand–receptor interactions of five inhibitors: (**a**) L24; (**b**) L28; (**c**) L30; (**d**) L33; (**e**) L35 at the active site of NUDT5. Amino acid residues involved in ligand interactions are highlighted, with interaction types shown in different colors.

Analysis of interactions between the selected ligands and the target protein (Figure 2 and Figure 3) revealed that they all formed hydrogen bonds and π–π stacking interactions with the key amino acid residues GluB 47 and TrpB 46, respectively. Ligand L1 formed hydrogen bonds with crucial amino acid residues TrpA 28 and ArgA 51. Inhibitors L30 and L35 formed an additional pi-sigma interaction with TrpB 46 and a van der Waals interaction with the prominent amino acid residue TrpA 28. Ligands L24, L28, and L33 established π–π stacking interactions with TrpA 28, while hydrogen bonds with ArgA 51. Additional hydrogen bonds and hydrophobic interactions were also observed, which further stabilize the ligands at the active site of NUDT5. Page et al. (2018) reported that interactions between NUDT5 inhibitors and the target protein are mainly formed through hydrogen bonds with ArgA 51 and GluB 47, as well as stacking interactions with TrpA 28 and TrpB 46 [24].

### 2.7. Correlation Between Binding Affinity Scores and Physicochemical Parameters

A correlation between binding affinity scores and various physicochemical parameters, namely Mr, nHBA, nHBD, logP, solubility, and Caco-2 permeability, was tested. A strong negative correlation between binding affinity and Mr (r = 0.802, *p* < 0.01), as well as a moderate correlation between binding affinity and nHBA (r = 0.581, *p* < 0.01), was observed. Furthermore, the correlation analysis revealed a statistically significant strong correlation (r = 0.698, *p* < 0.01) between binding free energy obtained by molecular docking and total binding free energy from MM/GBSA calculations.

## 3. Discussion

In women, BRCA is the most frequently diagnosed cancer and the leading cause of cancer-related death [29]. Considering the large impact on the female population, this disease is a public health concern, which requires a complete understanding of the molecular mechanism of BRCA, along with the improvement of the existing treatment. The complex nature of this disease, metastatic potential, and resistance to treatment are the main reasons for the failure of BRCA treatment [30]. The role of the NUDT enzymes has been well studied in BRCA. Due to its significant contribution to pathogenesis, NUDT5 appears to be a promising target for cancer therapy, making the identification of NUDT5 inhibitors crucial for potential treatment advances. Phytoactives and their derivatives are considered safer than synthetic substances in combating chemoresistance [9]. In silico analysis in this study identifies quercetin and its structural analogs as potential therapeutic agents for BRCA. Their inhibitory effects on NUDT5 protein structure and favorable pharmacokinetic profiles suggest their suitability for further development of anti-BRCA agents.

Analysis of ligand binding interaction by molecular docking is a well-known method of drug discovery worldwide. The results obtained from this study would help understand the inhibitory mode and rapidly and accurately predict the activities of new inhibitors based on docking score. Also, an essential aspect of molecular docking involves identifying the active site on the receptor. The active site refers to the specific region on the surface of the receptor where ligands bind, causing conformational changes that result in a pharmacological effect [31]. The information about the NUDT5 active site obtained in this study provides insights into how quercetin and its structural analogs achieve their anti-BRCA effect by inhibiting the NUDT5 receptor. Analyzing the results of molecular docking shown in Table 1 and Table S2, it is observable that all the tested ligands interact with the NUDT5 macromolecule. By comparing the binding energies of different compounds tested, it is notable that ligand L1 had the lowest binding energy, suggesting it to be the best binder of the target protein. Moreover, ligand L1 was the only one displaying a lower binding energy than the control inhibitor (co-crystallized ligand). However, it does not comply with the drug-likeness criteria of Lipinski’s rule and is less likely to be orally bioavailable. For ligands L1–L34, lower binding energy values were obtained in comparison to that of L35, indicating the stronger NUDT5 inhibitory potential of these compounds. The most common values for the selection of potential candidates that are currently accepted in drug design are those below −6.0 kcal/mol for binding free energy. However, there is still no consensus on the range that binding energies should fall within for biologically active compounds [32,33]. In our study, binding free energy values of all ligands were below the accepted limit of −6.0 kcal/mol. To further validate the molecular docking results, MM/GBSA binding free energy calculations were performed, which showed good correlation (r = 0.698, *p* < 0.01) with the docking scores.

Previous studies by other researchers have also examined the NUDT5 inhibitory potential of selected ligands to discover and develop new drugs for BRCA treatment. Using an in silico approach, Ruswanto et al. (2022) [34] demonstrated the possibility of developing thiourea compounds as therapeutic candidates for BRCA treatment through NUDT5 inhibition. A comparison of the binding energy values obtained using AutoDock for the ligands in our study and those in Ruswanto et al. (2022) shows that selected ligands in our research exhibit higher NUDT5 inhibitory activity due to achieving lower values of binding energy [34]. Furthermore, some ligands in our study achieve lower binding energy values than the imidazole derivatives investigated by Rashid et al. (2023), who used AutoDock to evaluate the NUDT5 inhibitory potential of these derivatives [35].

Acquiring detailed information about the interactions between ligands and macromolecules is a prerequisite for elucidating the nature, behavior, and activity of the formed complexes. Amino acid residues of proteins show a tendency to form hydrogen and van der Waals interactions [36]. The hydrogen bond is very common in the formation of ligand-macromolecule complexes [37], and the stronger it is, the more the complex approaches a more perfect geometry [31]. The studied compounds established hydrogen bonds, van der Waals forces and various hydrophobic interactions with key amino acid residues at the active site of NUDT5, which play an important role in the stability of the formed ligand-macromolecule complex. It is notable from Table 2 that none of the hydrogen bonds formed within the selected complexes fall under the strong category. L1 achieves the highest number of hydrogen bonds during complex formation, with two moderate and seven weak, and it is also the ligand that shows the highest affinity for NUDT5 inhibition. Most of the formed hydrogen bonds are weak. However, L28, L30, L35, and R form moderate hydrogen bonds in addition to weak ones. Although there is a tendency to consider only strong hydrogen bonds, this view can be misleading. Neglecting the weaker hydrogen bonds can lead to a complete failure to understand the static structure, and the only way to understand biological function is to consider both strong and weak hydrogen bonds together [38].

A study by Page et al. (2018) identified TrpA:28, ArgA:51, TrpB:46, and GluB:47 as key amino acid residues at the active site of the NUDT5 enzyme through which receptor-ligand interactions occur [24]. Tong et al. (2020) investigated the NUDT5 inhibitory activity of drugs approved for other indications (antipsychotics, antimicrobials, antiallergics, and antihypertensives) and found that those drugs that interacted with key residues TrpA:28 and TrpB:46 at the active site of NUDT5 suppressed BRCA cell viability to a greater extent, implicating the crucial role of these residues in NUDT5 [14].

The “rule of thumb” for drug-like property evaluation, also known as Lipinski’s rule of five, is a generally accepted method for predicting ADMET properties [39]. The rule of five aims to guide the discovery of small-molecule drugs that can be absorbed orally. Compounds with molecular properties showing not more than one violation of these rules are more likely to be orally available and exhibit lower failure rates throughout clinical trials [40]. In our study, eleven ligands out of thirty-six, including C, violate more than one of Lipinski’s rules. These ligands are less likely to be orally bioavailable. Although most drugs used in cancer treatment are given intravenously, the oral route of drug administration is the one preferred by patients. Around 50% of the FDA-approved small-molecule drugs either violate the rule of five or are not used orally. Therefore, in order to avoid overlooking potential lead molecules, Lipinski’s rules should be considered as guidelines rather than strict rules [41].

In silico assessment of pharmacokinetic properties plays a key role in selecting potential compounds for experimental studies [42]. Appreciation of the importance of ADMET properties has led to their consideration in the early stage of drug development, leading to a significant reduction in the number of compounds that failed in clinical trials due to poor ADMET properties [43]. An ideal oral drug should be absorbed from the gastrointestinal tract, distributed to the target specifically, metabolized without eliminating its properties, and removed without any damage. In this study, in silico ADMET tests enable the prediction of the ability of ligands to reach the target site of action—the NUDT5 receptor—in sufficient concentrations to produce an effective and safe pharmacological effect in the organism. Moreover, this information improves the quality of the drug candidate, increases the chances of successful clinical trials, facilitates the assessment of its toxicological profile, and helps reduce overall costs [44,45]. Based on the results of ADMET profiling, the most pronounced difference among the selected ligands was in their intestinal absorption, volume of distribution, metabolism, and total clearance. Metabolism is an important factor when considering drug interactions. The cytochrome P450 (CYP) superfamily is a key enzyme system for drug metabolism in the liver [27]. Inhibition of CYP enzymes hinders drug biotransformation or elimination, leading to higher plasma drug levels, possible toxicity, or lack of drug efficacy [26]. Among the human CYP enzymes, CYP1A2, 2B6, 2C8, 2C9, 2C19, 2D6, 3A4, and 3A5 are known to be responsible for the metabolism of the majority of drugs. CYP3A4 and 2D6 are the two main subtypes of CYP enzymes, contributing to over 50% of CYP-related drug metabolism [46,47]. CYPs can be inhibited or induced by concomitant drugs and circulating metabolites, which can influence treatment outcomes through drug–drug interaction. Understanding the role of specific CYP enzymes in a drug’s metabolism will help assess the likelihood and risk of CYP-mediated drug–drug interactions [47,48]. Some examples of anti-cancer drugs metabolized by CYP enzymes include tamoxifen by CYP2D6, docetaxel and cyclophosphamide by CYP3A4/5, and paclitaxel by CYP2C8 [47].

The results of ADMET analysis indicate that none of the compounds were a substrate or inhibitor of CYP2D6. Eight of the selected ligands were substrates and inhibitors of CYP3A4, while three (L1, L10, and L33) were substrates for CYP3A4, indicating the possibility of interactions with drugs that are metabolized by CYP3A4.

Results from toxicity analysis demonstrated that ligands categorized into toxicity classes four and five have high LD50 values, suggesting a potentially wide therapeutic window, i.e., a wider margin between the effective and the toxic dose. However, it is crucial to conduct careful dose-ranging studies to identify the optimal therapeutic dose needed for anti-cancer effects that maximize efficacy while minimizing toxicity.

Verifying the selectivity of a compound for the target protein over similar proteins is essential for optimizing its efficacy and safety to prevent off-target interactions that can lead to unwanted side effects, such as toxicity or unintended modulation of biological pathways. Non-selective binding to structurally similar proteins may disrupt normal cellular functions, causing adverse effects. Also, off-target interactions can diminish a compound’s therapeutic efficacy by engaging unintended biological pathways, resulting in unpredictable or counterproductive outcomes [13]. Ensuring selectivity helps to minimize the risk of adverse drug–drug interactions, especially in patients on multiple medications.

Based on in silico findings, several structural modifications of quercetin were observed and are suggested to improve the NUDT5 inhibitory activity of compounds. By replacing the catechol structure with a benzodioxin ring, the rigidity of the molecule could be increased, improving target specificity. Substitution of the benzodioxin ring at position 2 (hydrophilic substituent, e.g., hydroxymethyl) and 3 (bulky substituent, e.g., 4-hydroxy-3-methoxyphenyl) enables hydrogen bonding and hydrophobic interactions with the enzyme’s active site. Reduction in the C2–C3 double bond of the benzopyran ring changes the electronic properties, stability, and rigidity of the molecule, enhancing its biological activity. Introducing bulky substituents at position 7 of the benzopyran ring provides steric bulk that could improve binding affinity. In summary, the Structure–Activity Relationship (SAR) analysis indicates that structural modifications of quercetin and its analogs, including increased molecular rigidity, introduction of hydrophilic and bulky substituents, and adjustment of the electronic and steric properties of the benzopyran core, are key for enhancing NUDT5 inhibitory activity. Such changes in the quercetin analogs enable the formation of hydrogen bonds, van der Waals interactions and hydrophobic interactions with key amino acid residues at the NUDT5 active site. These interactions play a crucial role in the formation and stabilization of the ligand–NUDT5 complex, resulting in lower binding energy values and improved target selectivity. The identified structural modifications could guide further lead optimization and development.

By integrating the results of molecular docking, MM/GBSA calculations, compliance with Lipinski rules, and ADMET profiling, the two most promising ligands, L1 and L28, were selected. Based on the molecular docking results, MM/GBSA calculations, and ADMET profiling, L1 was identified as a promising NUDT5 inhibitor. Despite its non-compliance with the rules of Lipinski, the compound’s oral activity could be improved by the appropriate drug delivery system, such as nanoparticles or liposomes. Among the ligands that demonstrated higher binding affinity than quercetin in the docking studies and complied with the rules of Lipinski, taking into consideration the high values of Caco-2 permeability and intestinal absorption as the most important absorption parameters, the feature that it is neither a substrate nor an inhibitor of CYP and has a high toxicity threshold, L28 is proposed as an orally active BRCA drug candidate with NUDT5 inhibitory potential. It is important to emphasize that these studies rely on in silico approaches, and to corroborate the promising preliminary findings derived from computational methods, further experimental research is mandated. The translation of in silico results into further in vitro and in vivo studies will facilitate the development of these ligands as promising drug candidates, or at least as prototypes for new therapeutic agents in the treatment of BRCA.

By experimentally validating their in silico results, numerous studies have shown that computational methods can enable the discovery of BRCA inhibitors. Using molecular docking, scientists demonstrated the ERɑ inhibitory potential of genistein and daidzein and confirmed their in silico results using an ERɑ competitor assay and a luciferase reporter gene assay. These studies confirmed the feasibility of computational methods for predicting the inhibitory potential of ligands [49]. In a different study, researchers used a combination of molecular docking and molecular simulation to show that hesperidin is a flexible and potent ligand for the MLC-1 receptor. In vitro studies demonstrated the ability of hesperidin to kill the MDA-MB-231 cell lines without significantly affecting the MCF10A cell lines. Therefore, hesperidin can be a promising compound for BRCA treatment as a drug or food additive [50]. In addition, molecular docking and in silico ADMET studies demonstrated that jaceidin, a flavonoid isolated from *Chiladenus montanus*, is a selective inhibitor of VEGF with excellent membrane permeability and oral bioavailability. An in vitro study confirmed its cytotoxicity in the MCF-7 BRCA human line [51].

Flavonoids possess the potential to enhance the efficacy of current BRCA therapies, and to reduce both recurrence and chemoresistance in patients [52]. Supporting this, Li et al. (2018) reported that quercetin improves the cytotoxic effects of doxorubicin, paclitaxel, and vincristine by downregulating a transcription factor highly expressed in BRCA that regulates genes involved in cell proliferation and drug resistance [53]. Further studies have demonstrated synergistic effects of quercetin in combination with docetaxel in MCF7-DR [54] and MDA-MB-231 cells [55], as well as with 5-fluorouracil in MDA-MB-231 [56] and MCF-7 cells [57]. In addition, a study by Xu et al. (2020) reported that administration of quercetin at high concentrations alongside tamoxifen results in significantly synergistic antiproliferative and proapoptotic effects [58].

While this study provides valuable insights into the potential effectiveness of quercetin analogs as anti-BRCA drug candidates, it has several limitations. In database screening, compound libraries are examined to identify small molecules anticipated to interact with the drug target [59]. The success of screening and the validity of the results depend on the accuracy of these databases [13]. However, their role is not to replace but to complement experimental research, allowing the selection of compounds from a large database that will be the most adequate for the selected target [59]. Molecular docking and ADMET predictions are based on computational models that may not fully represent the complexity of biological systems. BRCA heterogeneity and inter-patient genetic variability are factors beyond the predictive capabilities of these in silico methods. Therefore, docking results must be validated through in vitro and in vivo assays to confirm the efficacy, specificity, and safety of the top hits. Standard docking protocols typically consider only a single ligand per binding site, which prevents the evaluation of potential synergistic effects between two or more molecules. In addition, molecular docking does not take into account the dynamic nature of proteins. This limitation could be addressed by employing molecular dynamics simulations to simulate the interaction between the ligands and the target protein over time. The study focused on quercetin and its 52 structural analogs. To expand the chemical space, a high-throughput virtual screening could be conducted on a broader set of quercetin analogs or other flavonoid compounds.

Although in silico methods are powerful tools in drug discovery, there is always the possibility of their improvement to increase their efficiency and precision. Future research that could improve knowledge on this topic should include a more complete determination of the pathogenesis of BRCA, along with an investigation of the interactions among its main participants. While molecular docking, MM/GBSA, and ADMET profiling provide valuable preliminary insights, molecular dynamics simulations could be employed to capture conformational changes and assess the stability of ligand–protein complexes over time, thereby improving the reliability of in silico predictions. Further steps should focus on bridging the gap between in silico predictions and clinical efficacy, given that favorable computational outcomes do not necessarily translate into therapeutic effectiveness. Promising candidates from in silico screening must be validated in vitro to assess their NUDT5 inhibitory activity and cytotoxic effects on BRCA cell lines. In vitro studies would allow the assessment of the specificity, potency, physicochemical properties, stability, and preliminary safety profile of the compounds. For promising candidates such as L1, the development of optimized drug delivery systems should be considered to improve bioavailability and pharmacokinetic properties. After validation and formulation, successful candidates will then progress to in vivo studies to evaluate their anti-cancer effects and ultimately into clinical trials to confirm or reject the potential for developing a new generation of drugs for BRCA treatment—NUDT5 inhibitors. In addition, combining NUDT5 inhibitors with existing chemotherapy or targeted therapies could be explored to enhance therapeutic outcomes. Expanding the scope of this work to include other cancer types that rely on NUDT5 activity may broaden the potential impact of these findings.

## 4. Materials and Methods

**Figure 4:**
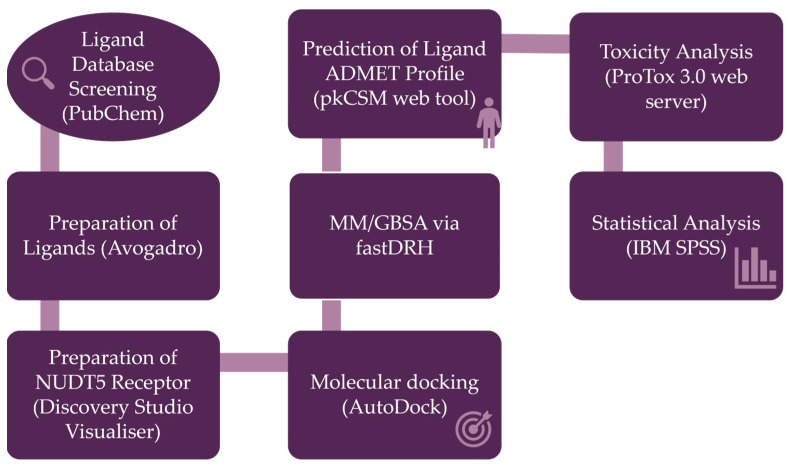
Study workflow illustrating the main methodological steps.

The workflow presented in Figure 4 summarizes the overall procedure of this study, from ligand selection to statistical analysis.

### 4.1. Preparation of Ligands for Molecular Docking

**Figure 5:**
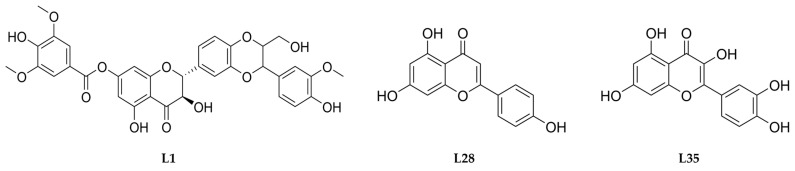
Chemical structures of ligands L1, L28, and L35.

Chemical structures of ligand molecules (L1-L53), two FDA-approved drugs (tamoxifen (T) and raloxifene (R)) used as positive controls, and the co-crystallized ligand (C): 7-[[5-(3,4-dichlorophenyl)-1,3,4-oxadiazol-2-yl]methyl]-1,3-dimethyl-8-piperazin-1-yl-purine-2,6-dione) were taken from the PubChem database (http://pubchem.ncbi.nlm.nih.gov/ accessed on 15 March 2025) in .sdf format [60]. The structures of molecules were geometry optimized using the software Avogadro 2.0 following the MMFF94 method [61]. Subsequently, .pdb files of the ligands were generated using Discovery Studio Visualiser 4.5 (DSV) (Dassault Systèmes BIOVIA, San Diego, CA, USA). Following ligand importation and root detection in the AutoDock Tools 1.5.7, the molecules were saved in .pdbqt format for molecular docking studies. The chemical structures of ligands L1, L28, and L35 are presented in Figure 5, while the structures of the remaining studied molecules are provided in Table S1 in the Supplementary File. Ligands were selected based on the chromene scaffold with a phenyl substituent at position 2, except for L47, which has the phenyl group at position 3. The ligands differ in their substitution patterns on both the chromene and phenyl rings, providing structural diversity for analysis.

### 4.2. Preparation of the NUDT5 Receptor for Docking

NUDT5 enzyme is crucial to cellular processes involved in nucleotide metabolism and cancer. Inhibition of NUDT5 can block nuclear ATP synthesis, hormone signaling, and cell proliferation in BRCA [24]. Therefore, in this computational study, it was selected as a target for anti-BRCA drug discovery. The three-dimensional structure of the NUDT5 receptor (PDB code: 5NWH) was downloaded from the Protein Data Bank (PDB) database (https://www.rcsb.org/ accessed on 15 March 2025) [24]. Using the DSV software package, ligand and water molecules were removed from the NUDT5-co-crystallized ligand complex. The AutoDock Tools 1.5.7 software package was used to assign polar hydrogen atoms and calculate charges using the Gasteiger method.

### 4.3. Molecular Docking Procedure

Molecular docking was performed using the AutoDock 4.2.6 software package (Molecular Graphics Laboratory, La Jolla, CA, USA), according to the standard procedure for a rigid receptor and a flexible ligand, with 25 independent repetitions per ligand. Ligand flexibility parameters, including the central atom (root) and the number of rotatable bonds, were automatically assigned by AutoDock software. The grid was set to a size of 60 points in the x, y, and z directions, and the distance between the grid points was 0.375 Å. The binding coordinates of the co-crystallized ligand were set as the grid center and were 12.294515, −14.443515, and −11.751455 in the x, y, and z directions, respectively. The specified coordinates were used during the docking of each ligand. The grid box files were saved as .gpf files, and the AutoGrid procedure was executed. The co-crystallized ligand (C) and two FDA-approved estrogen receptor-positive BRCA drugs—tamoxifen (T) and raloxifene (R)—were used as positive controls. Docking was performed using the Lamarckian Genetic Algorithm method [62]. The other parameters were set to default. Accordingly, the .dpf file for each ligand was generated and executed using the AutoDock procedure, and the resulting .dlg files were analyzed to obtain the values of binding energies (ΔGb) and the most favorable ligand–receptor conformations. The conformation with the lowest ΔGb was considered the most favorable. Visualization of the results and display of the types of interactions between compound and protein were performed using the DSV. In addition, DSV enabled the determination of amino acid residues that participate in the formation of hydrogen bonds and the distance and angle of hydrogen bonds between the formed complexes.

The docking procedure was validated by removing the co-crystallized ligand from the complex with NUDT5 and re-docking it to the receptor site using AutoDock 4.2.6. Root mean square deviation (RMSD) calculation included heavy atoms only. The RMSD between the obtained conformation and the original structure being less than 2 Å (0.367 Å) indicated the reliability of the binding ability prediction of new ligands [14].

### 4.4. Binding Free Energy Calculations

Molecular docking methods may produce false-positive results due to their inadequate consideration of receptor dynamics. Therefore, the validation of docking results through post-docking methods, such as MM/GBSA, provides a more comprehensive evaluation by incorporating solvation effects and entropic contributions. This approach ensures a more reliable and accurate estimation of the binding affinities of the investigated compounds [63]. The binding free energy for the complexes was calculated using fastDRH [64], an online web server, by submitting the Cartesian coordinates of the target receptor and the ligand molecules. For binding free energy calculation, the GB8 MM/GBSA procedure based on the pose ranking was used.

### 4.5. Determination of the Key Parameters of Lipinski’s Rule of Five

The significant parameters of Lipinski’s rule of five: Mr [g/mol], nHBA, nHBD, and logP were determined by the SwissADME web tool (http://www.swissadme.ch/ accessed on 20 March 2025). It gives free access to a group of fast, robust models for predicting physicochemical properties, pharmacokinetics, and drug-likeness for small molecules [65].

### 4.6. Prediction of ADMET Profile

ADMET properties of the docked ligands were predicted using the pkCSM web tool (https://biosig.lab.uq.edu.au/pkcsm/ accessed on 20 March 2025) [43].

### 4.7. Toxicity Analysis

The toxicity profile of the top-performing compounds (L1–L15) based on molecular docking results and MM/GBSA calculations was assessed using the ProTox 3.0 web server [66]. ProTox 3.0 employs molecular similarity and machine learning techniques to predict various toxicity endpoints for the analyzed compounds. Toxicity predictions included acute toxicity, organ toxicity, interactions with various receptors of the nuclear signaling pathway, interactions with proteins related to stress response pathways, and molecular initiating events.

### 4.8. Statistical Analysis

The correlation analysis between binding affinity scores and various physicochemical parameters was performed by IBM SPSS, version 22, and was reported as Pearson’s coefficient. A statistically significant correlation was determined with a level of significance of *p* < 0.05.

## 5. Conclusions

Despite advances in BRCA treatment, it remains a serious threat to individuals’ physical and mental health, as reflected by high mortality rates. Phytochemicals may provide therapeutic benefits when used alone or together with established anti-BRCA therapies as adjuvants. This study highlights the role of quercetin and its structural analogs in targeting the NUDT5 enzyme and their potential in anti-BRCA drug development. Novel insights into the NUDT5 inhibitory potential of quercetin and its structural analogs were provided through various computational techniques, including molecular docking, MM/GBSA, and ADMET analysis. Molecular docking simulations identified crucial binding interactions between the compounds and the NUDT5 active site, revealing that ligand inhibitory activity is primarily mediated through interactions with four key amino acid residues: Trp28 and Arg51 of chain A and Trp46 and Glu47 of chain B. Quercetin and its structural analogs with higher docking scores than quercetin were subjected to MM/GBSA calculations to support the binding affinity predictions further. Additionally, ADMET profiling enabled the selection of compounds possessing favorable pharmacokinetic properties. The comprehensive computational approach identified ligand L1 as the most promising candidate, suitable for delivery via an appropriate drug delivery system, while L28 emerged as a potential orally administered compound, both supporting further experimental validation. These results support the development of NUDT5-targeted therapeutics as a novel strategy in anti-BRCA research.

## Figures and Tables

**Table 1:** Results of molecular docking analysis of quercetin (L35) and structural analogs (L1–L34) having lower binding energies than quercetin (<−8.0 kcal/mol). Amino acid residues involved in hydrogen bonds and van der Waals interactions are indicated, highlighting their importance in stabilizing the NUDT5–ligand complex. ΔG values for L1 and L28, the ligands later identified as the most promising drug candidates, and for quercetin (L35) are shown in red.

Ligand	ΔGb (kcal/mol)	Interactions	
		Hydrogen Bond	Van Der Waals Interactions
L1	−11.24	LysA:161, AspA:164, TyrB:36, ArgB:44, LysA:27, TrpA:28, GluB:47, GlyB:135, ArgA:51	GlyA:165, GluA:115, ThrB:45, GluA:112, ValA:29, AlaA:96, LeuB:136
L2	−10.92	ArgA:196, TyrB:36, ArgA:51	LysA:161, AspA:164, GlyA:165, AspA:194, AlaA:63, AspB:133, GluA:166, ArgB:44, ThrB:45, GlnA:82, LeuB:136, GluB:47
L3	−10.76	AspA:194, GlyA:165, TyrB:36, ArgA:51	PheA:94, ArgA:196, ArgB:44, GluA:166, GlyA:97, ArgA:84, GluB:47, LeuB:136, ValA:29
L4	−10.42	ArgA:51, LeuA:98, GluA:112, LysA:161, AspA:164, GluB:47	LeuB:136, GlyB:135, GlyA:97, ArgA:111, ThrB:45, GluA:116
L5	−10.28	TyrB:36, GluB:47	ArgA:196, AspA:164, GlyA:165, ValA:62, GluA:166, GluA:93, GlyA:97, ArgA:84, LeuB:136, ValA:29
L6	−10.09	GlyA:165, TrpA:28, PheA:167, LysA:28, GluB:47, GlnB:15, PheA:83	ArgB:44, GluA:166, GlnA:82, LysB:33, ArgA:84
L7	−9.73	AlaA:96, ArgA:51, GlyB:135, PheA167, TrpA:28, GluB:47	GlyA:97, ArgA:84, GlnA:82, TrpB:46, ValA:168, GluA:166, LeuB:136, LysA:27, ValA:29, GlnB:15, AspB:38, ThrB:45, ProB:39
L8	−9.69	LeuA:98, GlyB:135, GluA:112, GluB:47, AspA:164, TrpB:46, ThrB:45	ArgA:84, LeuB:136, ArgA:111, ArgA:51, GluA:166, TrpA:28, GlyA:165, LysA:27
L9	−9.69	GluA:112, GluB:47, AspA:164	ArgA:111, LysA:161, ThrB:45
L10	−9.68	ArgB:44, LysA:27, TrpA:28, LeuA:98, TrpB:46, ArgA:51, GlyB:135, GluB:47	LysA:161, AspA:164, GlyA:165, ThrB:45, GluA:166, GluA:116, ArgA:111, GlyA:97, LeuB:136
L11	−9.65	ArgA:84, ArgA:51, GluA:115, GluB:47, TyrB:36, ThrB:45	GlnA:82, ArgA:111, GlyB:135, AlaA:96, GlyA:97, GluA:112, ValA:29, LeuB:136, LysA:161, LeuA:98, GluA:166, LysA:27, ArgB:44
L12	−9.64	ArgA:51, ThrB:45, AlaA:96, ArgA:84	ValA:29, GlyB:135, LeuB:136, GluB:47, TrpA:28, AspA:60, GlyA:61, GlyA:97, GluA:116, GlnA:82
L13	−9.53	GluA:166, ThrB:45, GlyB:135, GluB:47	ArgB:44, ArgA:84, TrpA:28, ValA:29
L14	−9.43	LeuA:98, GluB:47	ValA:62, CysA:139, LeuB:136, GlyA:97, GlyB:135, ArgA:196, AspB:133
L15	−9.42	GluB:47, ArgA:196, AspA:194	ValA:62, GlyA:97, IleA:141, LeuB:136, PheA:94, AspB:133, ArgB:44
L16	−9.42	GluA:112, GluB:47, AspA:164, ThrB:45	ArgA:111, GlyB:135, LeuB:136, TyrB:36, GluA:166
L17	−9.38	ArgA:51, GlyB:135, LeuA:98, ArgA:111, TrpB:46, GluB:47	GluA:115, ArgA:84, GluA:112, LeuB:136, GlyA:97, AspA:101, ThrA:53, LysA:27
L18	−9.34	LeuA:98, GluB:47, ArgA:51, AspA:164	ArgA:111, GlyA:97, GluA:112, ThrB:45, LysA:161, GluA:116, ValA:29, LeuB:136
L19	−9.19	GluB:47, GluA:115, GluA:112, LeuA:98, AlaA:96, ArgA:84	ValA:29, LeuB:136, TyrB:36, GlyB:135, IleA:99, GlyA:97, LysA:27, GlnA:82, GluA:116
L20	−8.87	AlaA:96, GlnA:82, ArgA:84, GluB:47, ThrB:45, ArgA:51, TyrB:36	GluA:116, SerB:48, ValA:29, LeuB:136, GluA:112, TrpA:28, TrpB:46, ArgB:44, GluA:166
L21	−8.82	GluA:166, TyrB:36, ArgA:51, ThrB:45, GluB:47	ArgA:84, GlyB:135, ArgB:44, LysA:27, LeuB:136, ValA:29, ValB:49, SerB:48, LysB:33
L22	−8.64	ThrB:45, GluB:47, ArgA:51	LysA:27, TyrB:36, ArgB:44, ValA:29, GluA:166, LeuB:136, SerB:48, GlyB:135
L23	−8.42	ThrB:45, GlyB:135, ArgA:51, GluB:47	ArgA:84, ArgB:44, ValA:29
L24	−8.41	GluB:47, ArgB:44, ArgA:51	LysA:27, ThrB:45, ArgA:84, LeuA:98
L25	−8.27	ArgA:51, GluB:47, ArgB:44	LeuA:98, ValA:29, LeuB:136, ThrB:45
L26	−8.21	GluB:47, ThrB:45, GlyB:135, ArgA:51	LeuA:98, ArgB:44, LeuB:136
L27	−8.15	ArgA:51, GluB:47, ThrB:45	LeuA:98, TyrB:36, GylB:135, SerB:48, LeuB:136, ValA:29, LysA:27
L28	−8.13	ArgA:51, ThrB:45, GluB:47	GlyB:135, LeuB:136, ValB:49, SerB:48, ValA:29, ArgB:44
L29	−8.08	ThrB:45, ArgA:51, GlyB:135	GluA:166, ArgA:84, GlyA:97, LysB:33, TrpA:28, GluB:47, LeuB:136
L30	−8.08	ThrB:45, GluB:47	ArgB:44, ArgA:51, GlyB:135, TrpA:28, VaA:29
L31	−8.06	GluB:47, ThrB:45, ArgA:51	ValA:29, LysA:27, SerB:48, LeuB:136, GlyB:135, ArgB:44
L32	−8.06	ThrB:45, GlyB:135, ArgA:51, gluB:47	ArgA:84, ArgB:44; TrpA:28, ValA:29
L33	−8.04	ArgA:51, ArgB:44, GluB:47	ArgA:84, GlyB:135, ThrB:45
L34	−8.01	ArgA:51, GluB:47, ThrB:45	TyrB:36, GlyB:135, LeuB:136, SerB:48, ValA:29
L35	−8.0	ThrB:45, GlyB:135, GluB:47	ArgB:44, ArgA:84, ValA:29
C	−11.02	GluB:47, ArgA:51	GluA:166, GlyB:135, LeuA:98, LeuB:136, GlyA:97, GlyA:61, ValA:62
T	−9.36	LeuA98	ArgA:84, GlyA:97, GluA:112, AlaA:96, GluB:47, ThrB:45, GlyB:135
R	−10.83	ArgA:84, ArgB:44, GluB:47	TyrB:36, LeuB:136, ValA:29, ArgA:51, GlyA:97, LeuA:98, ArgA:111, GluA:115, GluA:112, AlaA:96, GluA:116

ΔGb—binding energy; C—co-crystallized ligand; T—tamoxifen; R—raloxifen.

**Table 2:** Amino acid residues involved in hydrogen bond formation, type, length, and angle of hydrogen bond for selected complexes. During the formation of the complex, moderate and weak hydrogen bonds are established.

Complex	Name	Types	Distance	Angle DHA
NUDT5-L1	A:LYS27:NZ—:L1:O11	Conventional Hydrogen Bond	2.9052	
	A:TRP28:NE1—:L1:O11	Conventional Hydrogen Bond	3.0263	
	A:ARG51:NH2—:L1:O4	Conventional Hydrogen Bond	2.6999	
	A:LYS161:NZ—:L1:O10	Conventional Hydrogen Bond	2.7842	
	B:ARG44:NH1—:L1:O14	Conventional Hydrogen Bond	3.0866	
	:L1:H66—A:ASP164:O	Conventional Hydrogen Bond	2.0592	146.072
	:L1:H61—B:GLY135:O	Conventional Hydrogen Bond	2.1563	152.734
	:L1:H65—B:GLU47:O	Conventional Hydrogen Bond	2.3719	112.251
	:L1:H72—B:TYR36:OH	Conventional Hydrogen Bond	3.0413	94.802
NUDT5-L2	A:ARG51:NH1—:L2:O4	Conventional Hydrogen Bond	3.2128	
	A:ARG196:NH2—:L2:O12	Conventional Hydrogen Bond	2.8131	
	B:TYR36:OH—:L2:O13	Conventional Hydrogen Bond	2.4423	
NUDT5-L3	A:ARG51:NH1—:L3:O4	Conventional Hydrogen Bond	3.0429	
	B:TYR36:OH—:L3:O11	Conventional Hydrogen Bond	2.6881	
	:L3:H64—A:ASP194:OD2	Conventional Hydrogen Bond	1.8865	116.528
	:L3:H71—A:GLY165:O	Conventional Hydrogen Bond	2.3005	153.343
NUDT5-L4	A:ARG51:NH1—:L4:O4	Conventional Hydrogen Bond	2.9590	
	A:LYS161:NZ—:L4:O8	Conventional Hydrogen Bond	2.7955	
	A:ASP164:N—:L4:O10	Conventional Hydrogen Bond	3.1628	
	B:GLU47:N—:L4:O7	Conventional Hydrogen Bond	3.1725	
	:L4:H54—A:LEU98:O	Conventional Hydrogen Bond	2.7405	95.676
	:L4:H54—A:GLU112:OE1	Conventional Hydrogen Bond	2.2999	94.878
NUDT5-L5	B:TYR36:OH—:L5:O11	Conventional Hydrogen Bond	2.7707	
	B:GLU47:N—:L5:O6	Conventional Hydrogen Bond	3.3795	
	B:GLU47:N—:L5:O7	Conventional Hydrogen Bond	2.6243	
NUDT5-L24	A:ARG51:NH1—:L24:O4	Conventional Hydrogen Bond	3.0627	
	B:ARG44:NH1—:L24:O7	Conventional Hydrogen Bond	3.0560	
	B:GLU47:N—:L24:O3	Conventional Hydrogen Bond	3.0000	
	B:GLU47:N—:L24:O5	Conventional Hydrogen Bond	3.2257	
NUDT5-L28	A:ARG51:NH1—:L28:O2	Conventional Hydrogen Bond	2.8998	
	A:ARG51:NH2—:L28:O2	Conventional Hydrogen Bond	3.1644	
	:L28:H30—B:THR45:OG1	Conventional Hydrogen Bond	1.9621	154.122
	:L28:H29—B:GLU47:O	Conventional Hydrogen Bond	1.7022	158.33
NUDT5-L30	B:GLU47:N—:L30:O1	Conventional Hydrogen Bond	3.2047	
	:L30:H29—B:GLU47:OE2	Conventional Hydrogen Bond	2.1768	148.949
	:L30:H28—B:THR45:O	Conventional Hydrogen Bond	2.0593	126.724
NUDT5-L33	A:ARG51:NH1—:L33:O3	Conventional Hydrogen Bond	2.9274	
	A:ARG51:NH2—:L33:O3	Conventional Hydrogen Bond	3.1588	
	B:ARG44:NH1—:L33:O6	Conventional Hydrogen Bond	3.0908	
	B:GLU47:N—:L33:O5	Conventional Hydrogen Bond	2.5814	
	:L33:H30—B:GLU47:O	Conventional Hydrogen Bond	2.4846	133.572
NUDT5-L35	B:GLU47:N—:Quercetin:O1	Conventional Hydrogen Bond	3.2024	
	:Quercetin:H31—B:GLY135:O	Conventional Hydrogen Bond	2.1313	131.074
	:Quercetin:H32—B:GLY135:O	Conventional Hydrogen Bond	2.1999	113.465
	:Quercetin:H30—B:GLU47:OE2	Conventional Hydrogen Bond	2.3163	146.64
	:Quercetin:H29—B:THR45:O	Conventional Hydrogen Bond	2.2237	129.379
NUDT5-C	A:ARG51:NH1—A:Control:O32	Conventional Hydrogen Bond	2.8128	
	B:GLU47:N—A:Control:O33	Conventional Hydrogen Bond	2.6402	
NUDT5-T	A:LEU98:N—:Tamoxifen:O1	Conventional Hydrogen Bond	3.0922	
NUDT5-R	A:ARG84:HH22—:Raloxifen:O2	Conventional Hydrogen Bond	2.4916	128.533
	B:ARG44:HH12—:Raloxifen:O5	Conventional Hydrogen Bond	2.0731	161.962
	:Raloxifen:H60—B:GLU47:O	Conventional Hydrogen Bond	2.0734	156.171

The classification of hydrogen bonds is crucial to understanding molecular interactions. Jeffrey (1997) [25] defined a strong hydrogen bond as one with a distance of 1.2–1.5 Ǻ and a D–H–A angle of 170–180° and a moderate hydrogen bond with a distance of 1.5–2.2 Ǻ, as well as a D–H–A angle of 130–180°. Also, he defined a weak hydrogen bond as one that has a distance > 2.2 Ǻ and an angle D–H–A ranging from 90° to 150° [25].

**Table 3:** MM/GBSA binding free energies determined for the selected complexes along with the energy terms: electrostatic contribution (ELE); van der Waals contribution (VDW); non-polar SASA contribution, and the polar contribution (Polar) in kcal/mol, showing their impact on overall binding affinity and complex stability. Total binding free energy values for L1 and L28, the ligands later identified as the most promising drug candidates, and for quercetin (L35) are highlighted in red.

Ligand	ELE	VDW	Non-Polar SASA	Polar	Total Binding Free Energy
L5	0	−75.66	−6.38	34.2	−47.83
L10	0	−83.53	−7.18	43.77	−46.94
L1	0	−74	−5.97	34.76	−45.21
L3	0	−71.18	−5.91	36.16	−40.93
L2	0	−76.37	−6.34	41.85	−40.86
L8	0	−59.17	−4.88	25.92	−38.13
L9	0	−61.5	−5.07	30.15	−36.41
L15	0	−61.47	−5.06	32.07	−34.46
L14	0	−54.43	−4.28	27.18	−31.53
L4	0	−51.3	−5.13	29.18	−27.24
L25	0	−41.7	−3.27	18.1	−26.86
L34	0	−41.88	−3.76	18.8	−26.84
L19	0	−53.12	−4.88	31.57	−26.43
L22	0	−43.53	−3.7	21.71	−25.51
L20	0	−58.42	−5.59	39.01	−25.01
L32	0	−34.7	−2.66	12.8	−24.55
L13	0	−49.16	−4.24	29.53	−23.86
L6	0	−45.23	−3.62	25.55	−23.3
L16	0	−51.38	−5.2	33.33	−23.26
L17	0	−49.22	−4.88	30.88	−23.23
L18	0	−55.68	−5.35	37.85	−23.18
L27	0	−36.55	−2.46	16.18	−22.84
L24	0	−41.24	−3.08	21.93	−22.38
L7	0	−58.32	−5.1	41.15	−22.27
L26	0	−38.29	−2.95	19.08	−22.16
L12	0	−53.7	−5.12	36.87	−21.96
L31	0	−34.57	−2.37	15.35	−21.57
L23	0	−38.45	−3.11	20.09	−21.45
L35	0	−33.63	−2.81	15.87	−20.58
L11	0	−58.5	−5.92	45.36	−19.06
L28	0	−39.05	−3.66	26.67	−16.04
L30	0	−37.1	−3.57	27.48	−13.2
L21	0	−52.77	−4.75	45.35	−12.16
L29	0	−39.67	−3.97	32.34	−11.31
L33	0	−34.9	−3.77	32.8	−5.88
C	0	−59.1	−3.96	16.91	−46.16
R	0	−55.19	−4.86	28.56	−31.51
T	0	−45.13	−3.98	21.87	−27.24

R—raloxifene; T—tamoxifen.

**Table 4:** Lipinski properties of docked ligands, water solubility, and Caco-2 permeability, summarizing the drug-likeness profile, aqueous solubility, and predicted intestinal permeability of the ligands, thereby providing insights into their potential as orally bioavailable drug candidates.

Ligand	Mr	nHBA	nHBD	logP	Number of Violations of Lipinski’s Rules	Solubility in Water (log mol/L)	Caco-2 Permeability [log cm/s]
	(g/mol)						
**L1**	662.59	14	5	2.82	2	−3.146	0.126
**L2**	684.64	13	4	3.95	2	−3.144	0.301
**L3**	634.54	14	7	2.05	3	−2.906	−0.93
L4	524.52	10	2	2.63	1	−4.104	0.848
**L5**	612.58	11	4	3.57	2	−3.394	0.246
L6	482.44	10	5	1.59	0	−3.204	0.435
**L7**	608.54	15	8	−0.44	3	−2.929	0.305
L8	482.44	10	5	1.59	0	−3.204	0.435
L9	496.46	10	4	1.97	0	−3.375	0.222
**L10**	658.6	13	5	3.18	2	−3.125	0.097
**L11**	580.53	14	8	−0.79	3	−2.919	−0.658
L12	466.44	9	4	1.84	0	−3.639	0.242
**L13**	448.38	11	7	0.16	2	−2.903	0.048
L14	524.52	10	2	2.91	1	−4.468	0.559
L15	496.46	10	4	1.97	0	−3.735	0.22
L16	480.42	10	5	2.33	0	−2.971	0.457
**L17**	538.54	10	1	3.12	1	−4.128	0.91
L18	482.44	10	5	1.59	0	−3.204	0.435
**L19**	612.53	16	10	−1.76	3	−2.897	−1.302
**L20**	652.6	18	8	−0.65	3	−2.978	0.291
**L21**	610.52	16	10	−1.29	3	−2.892	−0.949
L22	370.35	7	5	2.69	0	−2.918	−0.107
L23	330.29	7	3	2.2	0	−3.241	0.387
**L24**	372.37	7	0	3.02	0	−4.792	1.245
L25	368.38	6	3	3.53	0	−3.92	0.352
L26	330.29	7	3	2.02	0	−3.399	−0.002
L27	300.26	6	3	2.19	0	−3.238	0.326
**L28**	270.24	5	3	2.11	0	−3.329	1.007
L29	354.35	6	4	3.22	0	−3.303	−0.185
**L30**	254.24	4	2	2.55	0	−3.538	0.945
L31	286.24	6	4	1.73	0	−3.094	0.096
L32	316.26	7	4	1.65	0	−3.000	−0.003
**L33**	314.29	6	2	2.46	0	−3.481	1.022
L34	302.28	6	3	1.91	0	−3.047	0.294
L35	302.24	7	5	1.23	0	−2.925	−0.229
C	491.33	7	1	1.99	0	−2.856	0.253
T	371.51	2	0	5.77	1	−5.929	1.065
R	459.56	5	2	5	0	−3.716	0.77

nHBA—number of hydrogen bond acceptors; nHBD—number of hydrogen bond donors; logP—logarithm of partition coefficient; T—tamoxifen; R—raloxifen. Ligands that violate more than one criterion of Lipinski’s rules are bolded, and those having the best values for Caco-2 permeability are marked in red.

**Table 5:** Toxicity profile of selected ligands and reference drugs, including LD_50_ values, toxicity class, and predicted organ-specific risks, along with carcinogenicity, immunotoxicity, mutagenicity, and cytotoxicity, thereby providing a comprehensive assessment of their safety potential.

Ligand	LD_50_ (mg/kg)	Toxicity Class	Hepatotoxicity	Neurotoxicity	Nephrotoxicity	Cardiotoxicity	Carcinogenicity	Immunotoxicity	Mutagenicity	Cytotoxicity
L1	2000	4	Inactive	Inactive	Active	Active	Inactive	Active	Inactive	Inactive
L2	25	2	Inactive	Inactive	Active	Active	Inactive	Active	Inactive	Inactive
L3	2000	4	Inactive	Inactive	Active	Active	Inactive	Active	Inactive	Inactive
L4	1000	4	Inactive	Inactive	Active	Active	Inactive	Active	Inactive	Inactive
L5	25	2	Inactive	Inactive	Active	Active	Inactive	Active	Inactive	Inactive
L8	2000	4	Inactive	Inactive	Active	Active	Inactive	Active	Inactive	Inactive
L9	2000	4	Inactive	Inactive	Active	Active	Inactive	Active	Inactive	Inactive
L10	25	2	Inactive	Inactive	Active	Active	Inactive	Active	Inactive	Inactive
L14	2000	4	Inactive	Inactive	Active	Active	Inactive	Active	Inactive	Inactive
L15	2000	4	Inactive	Inactive	Active	Active	Inactive	Active	Inactive	Inactive
L17	1000	4	Inactive	Inactive	Active	Active	Inactive	Inactive	Inactive	Inactive
L24	5000	5	Inactive	Inactive	Active	Inactive	Inactive	Inactive	Inactive	Inactive
L28	2500	5	Inactive	Inactive	Active	Inactive	Inactive	Inactive	Inactive	Inactive
L30	3919	5	Inactive	Inactive	Active	Inactive	Inactive	Inactive	Inactive	Inactive
L33	4000	5	Inactive	Inactive	Active	Inactive	Inactive	Inactive	Inactive	Inactive
L35	159	3	Inactive	Inactive	Active	Inactive	Active	Inactive	Active	Inactive
C	684	4	Inactive	Active	Inactive	Inactive	Inactive	Inactive	Active	Inactive
T	1190	4	Active	Active	Inactive	Inactive	Inactive	Active	Inactive	Inactive
R	1000	4	Inactive	Active	Active	Inactive	Inactive	Inactive	Inactive	Inactive

## Data Availability

Data will be made available on request.

## Supplementary Materials

The following supporting information can be downloaded at: https://www.mdpi.com/article/10.3390/ijms26188843/s1.
