# Supplementary material for: Quercetin and Its Structural Analogs as NUDT5 Inhibitors: A Preliminary In Silico Study
Source: Int J Mol Sci. 2025 Sep 11;26(18):8843. doi: 10.3390/ijms26188843 (PMC12469961; doi:10.3390/ijms26188843)
Supplement: Supplementary file 1 [file ijms-26-08843-s001.zip › ijms-3830916-supplementary.pdf]

# Supplementary material

Quercetin and Its Structural Analogs as NUDT5 Inhibitors: A Preliminary In Silico Study

**Emilia Gligorić *, Milica Vidić *, Branislava Teofilović and Nevena Grujić-Letić**

Department of Pharmacy, Faculty of Medicine, University of Novi Sad, Hajduk Veljkova 3, 21000 Novi Sad, Serbia; branislava.teofilovic@mf.uns.ac.rs (B.T.); nevena.grujic-letic@mf.uns.ac.rs (N.G.-L.)

* Correspondence: emilia.gligoric@mf.uns.ac.rs (E.G.); milica.vidic@mf.uns.ac.rs (M.V.); Tel.: +381-214-227-60 (E.G.)

**Table S1** Chemical structures of ligand molecules

| Molecule | Chemical structure |
|---|---|
| L1 | 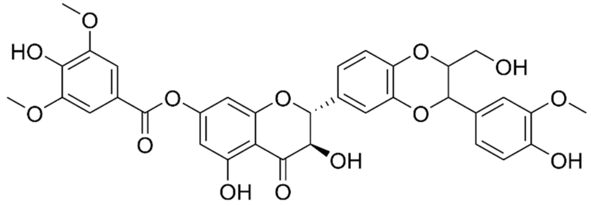 |
| L2 | 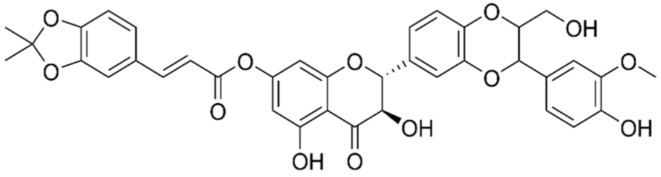 |
| L3 | 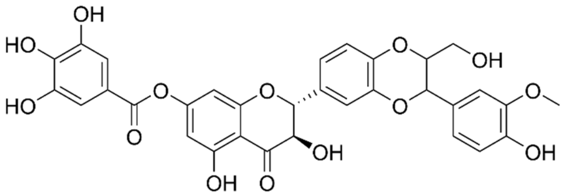 |
| L4 | 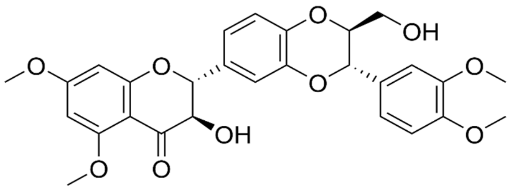 |
| L5 | 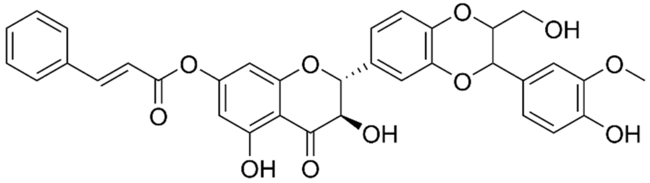 |

L6

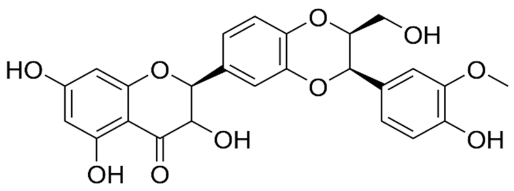


L7

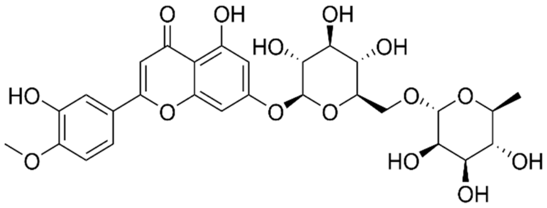


L8

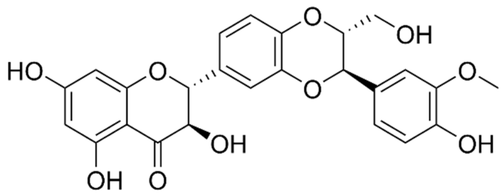

L9

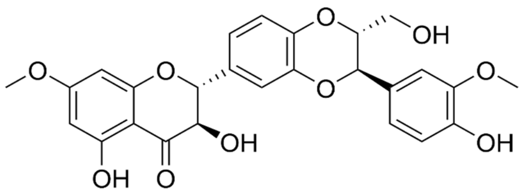

L10

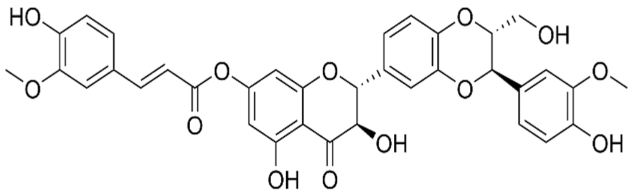

L11

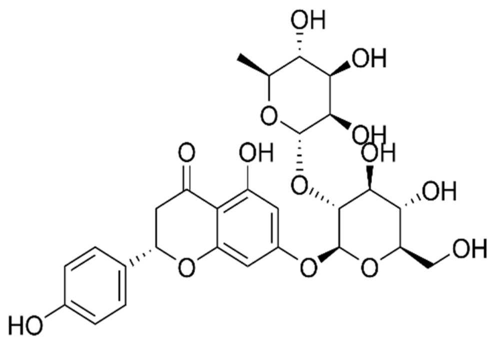


L12

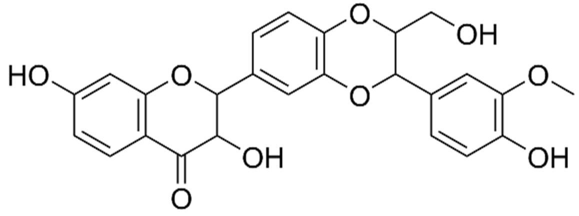


L13

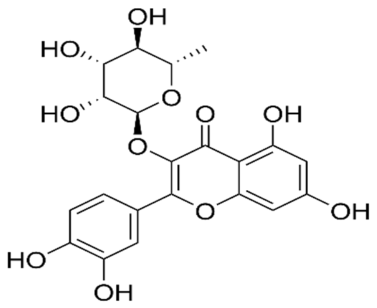


L14

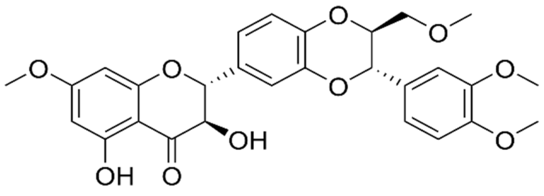

L15

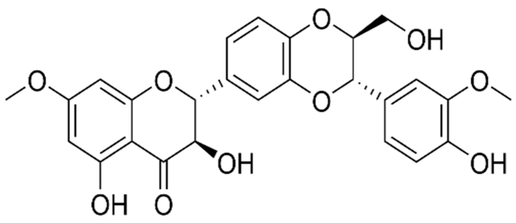

L16

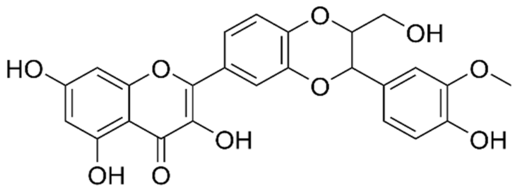

L17

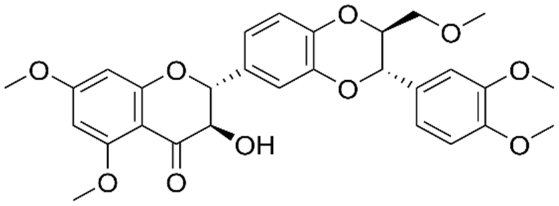

L18

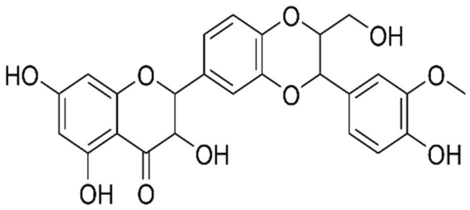

L19

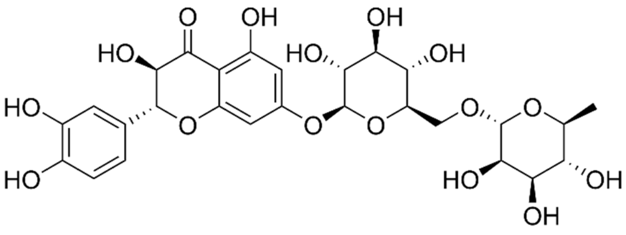

L20

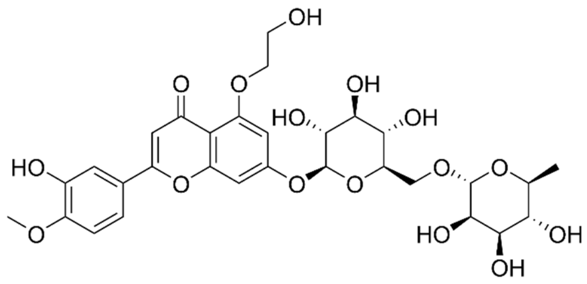

L21

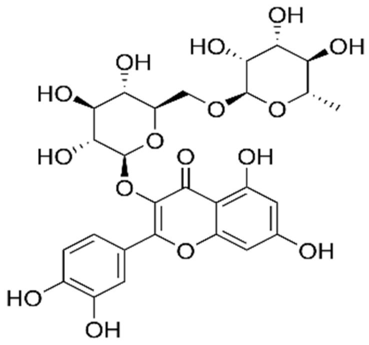

L22

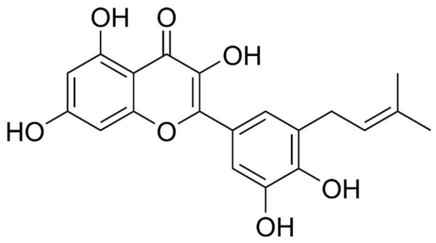

L23

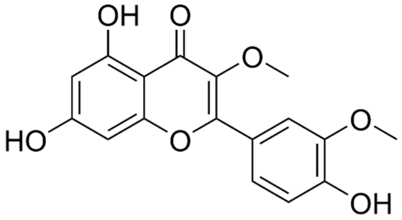

L24

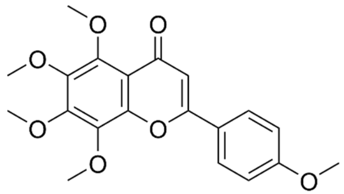


L25

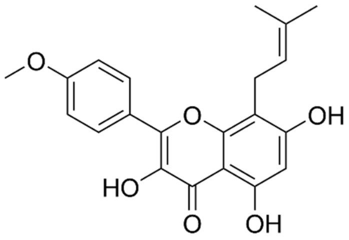


L26

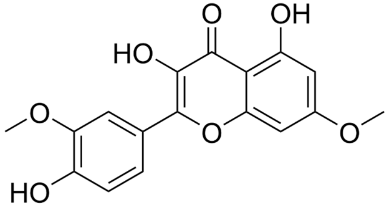


L27

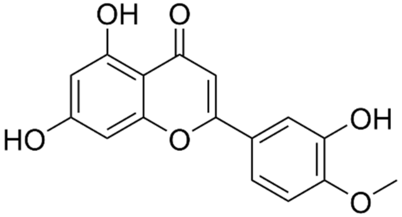


L28

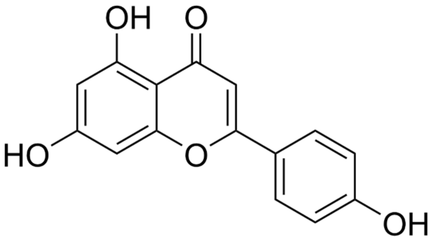

L29

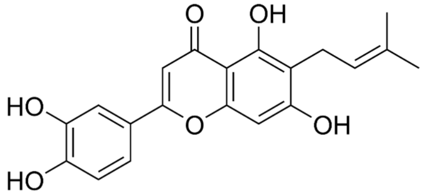


L30

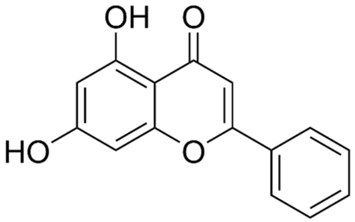


L31

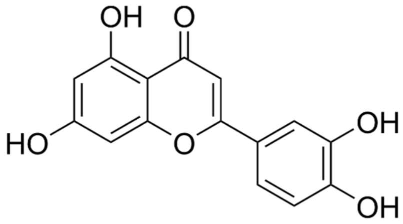


L32

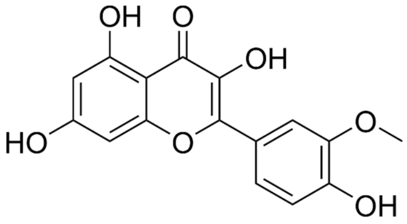


L33

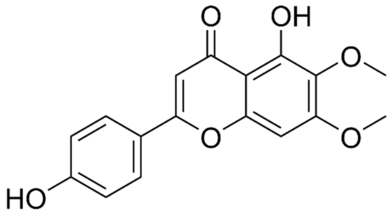

L34

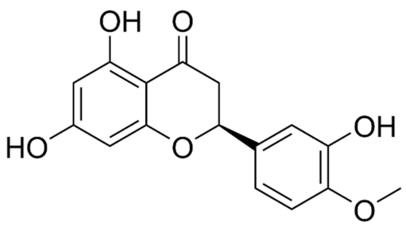


L35

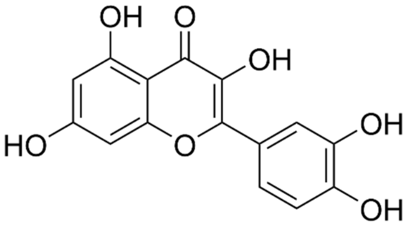


L36

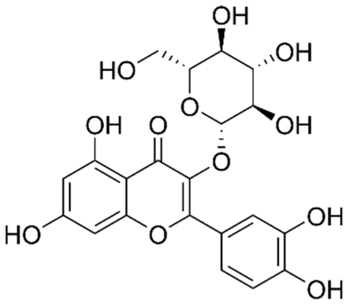


L37

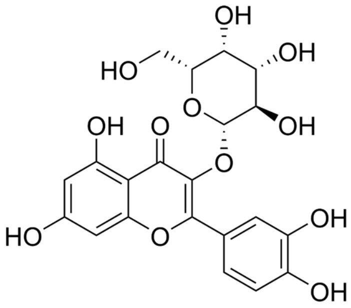

L38

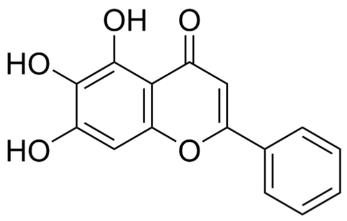


L39

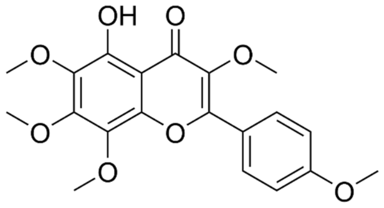


L40

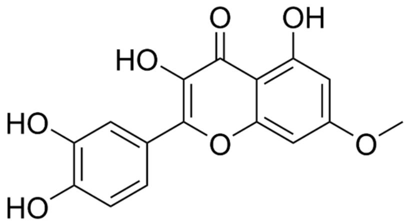


L41

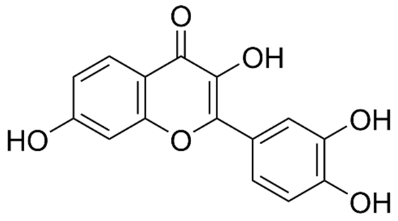


L42

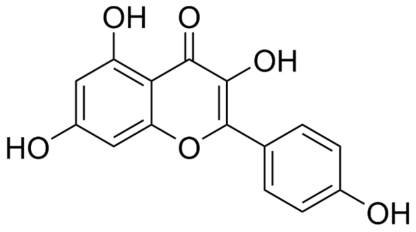

L43

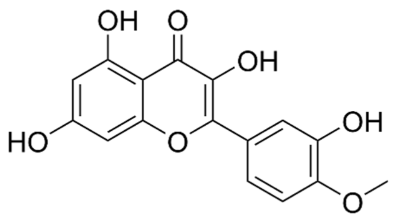


L44

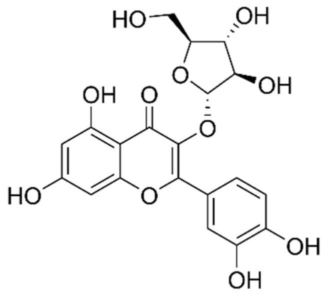


L45

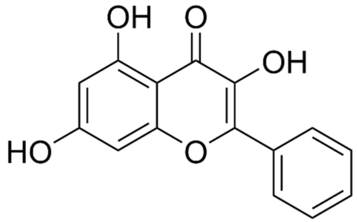


L46

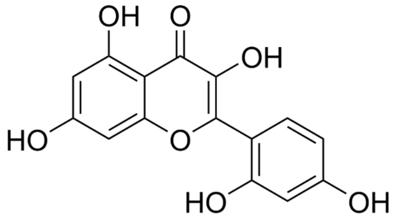

L47 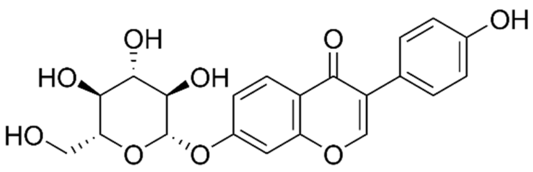


L48 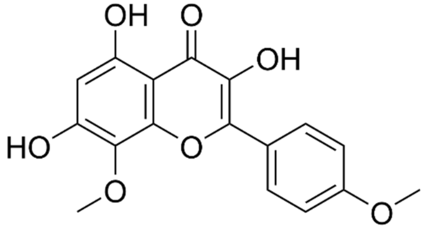


L49 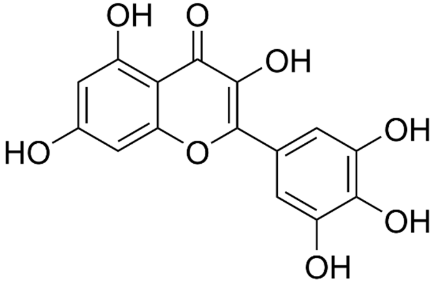


L50 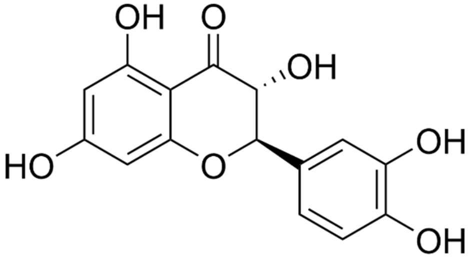

L51

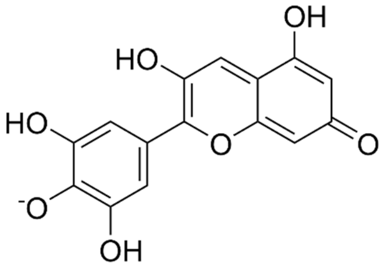
OH
HO
HO
O
O
-O
OH

L52

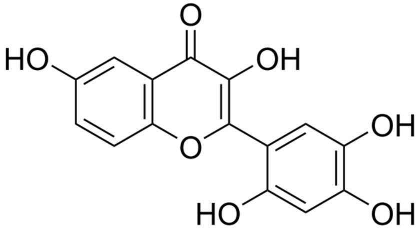
O
HO
OH
OH
O
HO
OH

L53

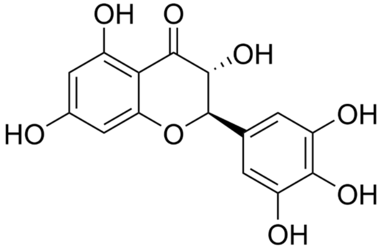
OH
O
OH
OH
HO
O
OH
OH

C

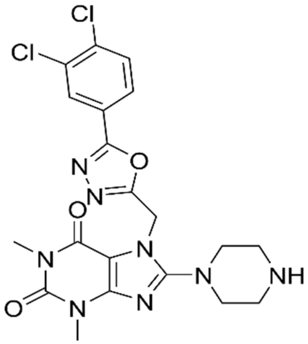
Cl
Cl
N
O
N
O
N
N
N
NH
O
N
N

T

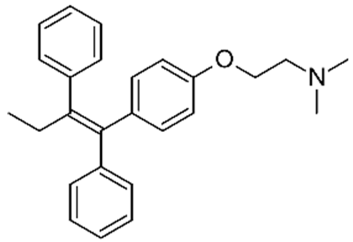


R

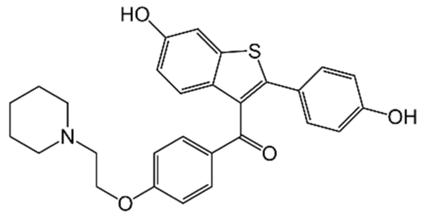


C, co-crystallized ligand from the complex pdb: 5nwh; T, tamoxifen; R, raloxifen

**Table S2** Results of docking analysis of ligands (L36-52) with binding energies higher than quercetin (>-8.0 kcal/mol) and NUDT5 macromolecule

| Ligand | ΔGb [a] (kcal/mol) | Interactions | |
|---|---|---|---|
| | | Hydrogen bond | Van der Waals interactions |
| L36 | -7.95 | GluA:166, GluB:47, ArgA:51 | TyrB:36, ThrB:45, ValA:29, SerB:48, LeuB:136, ValB:49, GlyA:97, ArgA:84 |
| L37 | -7.93 | GluA:166, ArgA:51, GlyB:135, ThrB:45, GluB:47 | LeuA:98, TyrB:36, ValA:29, LysB:33 |
| L38 | -7.93 | GluB:47, GlyB:135 | LysB:33, TrpA:28, ArgA:51, LeuB:136 |
| L39 | -7.88 | ArgA:51, ArgB:44, GluB:47 | LeuA:98, ThrB:45, LysA:27 |
| L40 | -7.86 | GluB:47, GlyB:135, ArgA:51, ThrB:45 | ValA:29, LeuB:136, TrpA:28, LysB:33, ArgA:84 |
| L41 | -7.86 | GlyB:47, GlyB:135 | ThrB:45, ArgA:84, TrpA:28, ValA:29 |
| L42 | -7.8 | ArgA:51, GluB:47, ThrB:45 | GlyB:135, LeuB:136, ArgB:44, ValA:29, SerB:48. LysA:27 |

| | | | |
|---|---|---|---|
| L43 | -7.8 | ArgA:51, ThrB:45, GluB:47 | TyrB:36, GlyB:135, LeuB:136, SerB:48, LysA:27, ValA:29 |
| L44 | -7.78 | GluB:47, ThrB:45, TyrB:36, ArgA:51, GlyB:135 | ValA:29, TrpA:28, LeuB:136, ArgB:44 |
| L45 | -7.78 | GluB:47, ThrB:45 | ValA:29, GlyB:135, ArgA:51, TrpA:28, ArgB:44 |
| L46 | -7.77 | GluB:47, ThrB:45, TyrB:36, ArgB:44, ArgA:51, GlyB:135 | TrpA:28, LeuB:136, GluA:166, GlyA:165 |
| L47 | -7.77 | LeuA:98, GluA:112, GluA:166, GluA:116, AlaA:96 | ArgA:51, GlyB:135, ThrB:45, ArgA:111, GlyA:97, GluB:47, TrpA:28, LysB:33; GluA:115 |
| L48 | -7.74 | ArgA:51, GluB:47 | GlyB:135, LeuB:136, ValA:29, TyrB:36 |
| L49 | -7.59 | ArgA:51, GluB:47, ThrB:45 | GlyB:135, TyrB:36, ValA:29, ArgB:44, LeuB:136, SerB:48, LysA:27 |

| | | | |
|---|---|---|---|
| L50 | -7.58 | ArgA:51, GluB:47, ThrB:45 | GlyB:135, LysA:27, LeuB:136,ValA:29, SerB:48, TyrB:36, ArgB:44 |
| L51 | -7.5 | TyrB:36, ArgA:51, ThrB:45, GluB:47 | ArgB:44, GlyB:135, LeuB:136, SerB:48, ValA:29, LysA:27 |
| L52 | -7.39 | TyrB:36, ThrB:45, GluB:47, GluA:146 | ArgB:44, LysA:27, TrpA:28, ValA:29, LeuB:136, GlyB:135, ArgA:51 |
| L53 | -7.36 | ArgA:51, ThrB:45, GluB:47 | LeuA:98, LysA:27, GlyB:135, LeuB:136, ValA:29, SerB:48, TyrB:36, ArgB:44 |

[a] $\Delta$Gb – binding energy

**Table S3** ADMET profile of selected ligands obtained by the pkCSM web tool

| ADMET parameters | Ligand molecules | | | | | | | | | | | | | | | | Reference compounds | | |
|---|---|---|---|---|---|---|---|---|---|---|---|---|---|---|---|---|---|---|---|
| | **L1** | **L2** | **L3** | **L4** | **L5** | **L8** | **L9** | **L10** | **L14** | **L15** | **L17** | **L24** | **L28** | **L30** | **L33** | **L35** | **T** | **R** | **C** |
| Intestinal absorption [%] | 82.33 | 93.35 | 50.2 | 79.18 | 79.66 | 61.86 | 84.24 | 77.36 | 52.71 | 100 | 96.67 | 98.47 | 93.25 | 93.76 | 93.98 | 77.20 | 96.88 | 93.52 | 78.88 |
| Volume of distribution [logL/kg] | -1.303 | -1.634 | -0.259 | -0.776 | -1.449 | 0.369 | 0.697 | -1.437 | 1.517 | -0.652 | -0.961 | -0.226 | 0.822 | 0.403 | 0.001 | 1.559 | 0.83 | -1.492 | 0.549 |
| Fraction unbound | 0.087 | 0.226 | 0.167 | 0.205 | 0.146 | 0 | 0 | 0.089 | 0.13 | 0.089 | 0.267 | 0.188 | 0.147 | 0.136 | 0.093 | 0.206 | 0.093 | 0.114 | 0.279 |
| Blood-brain barrier (BBB) permeability [logBB] | -2.046 | -1.856 | -2.339 | -1.373 | -1.534 | -1.207 | -1.375 | -1.721 | -1.495 | -1.269 | -1.515 | -1.026 | -0.73 | 0.047 | -0.59 | -1.098 | 1.329 | -1.039 | -2.137 |
| CYP2D6 substrate | No | No | No | No | No | No | No | No | No | No | No | No | No | No | No | No | No | No | No |
| CYP3A4 substrate | Yes | Yes | No | Yes | Yes | No | Yes | Yes | No | Yes | Yes | Yes | No | No | Yes | No | Yes | Yes | Yes |
| CYP2D6 inhibitior | No | No | No | No | No | No | No | No | No | No | No | No | No | No | No | No | Yes | No | No |
| CYP3A4 inhibitior | No | Yes | No | Yes | Yes | No | Yes | No | No | Yes | Yes | Yes | No | No | No | No | No | Yes | Yes |
| Total Clearance [ml/min/kg] | -0.647 | -1.031 | -0.925 | 0.161 | -0.254 | -0.103 | -0.062 | -0.51 | 0.364 | 0.227 | 0.318 | 0.78 | 0.566 | 0.405 | 0.587 | 0.407 | 0.556 | 0.746 | 0.109 |
| AMES toxicity | No | No | No | No | No | No | No | No | No | No | No | No | No | No | No | No | Yes | No | Yes |
| Maximum tolerated | 0.398 | 0.354 | 0.459 | 0.481 | 0.328 | 0.65 | 0.244 | 0.397 | 0.495 | 0.233 | 0.556 | 0.385 | 0.328 | 0.016 | 0.033 | 0.499 | 0.313 | 0.161 | 0.555 |

| | | | | | | | | | | | | | | | | | | | |
|---|---|---|---|---|---|---|---|---|---|---|---|---|---|---|---|---|---|---|---|
| dose [mg/kg/day] | | | | | | | | | | | | | | | | | | | |
| Oral Rat Acute Toxicity [mol/kg], $LD_{50}$ | 2.754 | 2.89 | 2.547 | 2.715 | 2.767 | 2.559 | 2.539 | 2.744 | 2.586 | 3.38 | 3.778 | 2.368 | 2.45 | 2.289 | 2.254 | 2.471 | 2.285 | 2.521 | 2.64 |
| Oral Rat Chronic Toxicity [mg/kg/day], LOAEL | 3.798 | 3.297 | 4.491 | 0.937 | 3.545 | 3.494 | 2.999 | 3.886 | 3.022 | 2.47 | 1.008 | 0.944 | 2.298 | 0.955 | 1.862 | 2.612 | 0.41 | 1.12 | 0.832 |
| Hepatotoxicity | No | No | No | No | No | No | No | No | No | No | No | No | No | No | No | No | No | Yes | Yes |

**Table S4** Interactions of selected ligands with various receptors of the nuclear signalling pathway

| Ligand | Nuclear receptor signalling pathways | | | | | | |
|---|---|---|---|---|---|---|---|
| | **Aryl hydrocarbon Receptor (AhR)** | **Androgen Receptor (AR)** | **Androgen Receptor Ligand Binding Domain (AR-LBD)** | **Aromatase** | **Estrogen Receptor Alpha (ER)** | **Estrogen Receptor Ligand Binding Domain (ER-LBD)** | **Peroxisome Proliferator Activated Receptor Gamma (PPAR-Gamma)** |
| **L1** | Inactive | Inactive | Inactive | Inactive | Inactive | Inactive | Inactive |
| **L2** | Inactive | Inactive | Inactive | Inactive | Inactive | Inactive | Inactive |
| **L3** | Active | Inactive | Inactive | Inactive | Inactive | Inactive | Inactive |
| **L4** | Active | Inactive | Inactive | Inactive | Inactive | Inactive | Inactive |
| **L5** | Inactive | Inactive | Inactive | Inactive | Inactive | Inactive | Inactive |
| **L8** | Active | Inactive | Inactive | Inactive | Inactive | Inactive | Inactive |
| **L9** | Active | Inactive | Inactive | Inactive | Inactive | Inactive | Inactive |
| **L10** | Active | Inactive | Inactive | Inactive | Inactive | Inactive | Inactive |
| **L14** | Active | Inactive | Inactive | Inactive | Inactive | Inactive | Inactive |
| **L15** | Active | Inactive | Inactive | Inactive | Inactive | Inactive | Inactive |
| **L17** | Active | Inactive | Inactive | Inactive | Inactive | Inactive | Inactive |
| **L24** | Active | Inactive | Inactive | Inactive | Active | Inactive | Inactive |
| **L28** | Active | Inactive | Inactive | Active | Active | Active | Active |

| | | | | | | | |
|---|---|---|---|---|---|---|---|
| **L30** | Active | Inactive | Inactive | Active | Active | Active | Active |
| **L33** | Active | Inactive | Inactive | Inactive | Active | Active | Inactive |
| **L35** | Active | Inactive | Inactive | Inactive | Active | Active | Inactive |
| **C** | Inactive | Inactive | Inactive | Inactive | Inactive | Inactive | Inactive |
| **Tamoxifen** | Inactive | Inactive | Inactive | Active | Active | Active | Inactive |
| **Raloxifen** | Inactive | Inactive | Inactive | Active | Inactive | Inactive | Inactive |

**Table S5** Interactions of selected ligands with proteins related to stress response pathways

| **Ligand** | **Stress response pathways** | | | | |
|---|---|---|---|---|---|
| | **Nuclear factor (erythroid-derived 2)-like 2/antioxidant responsive element (nrf2/ARE)** | **Heat shock factor response element (HSE)** | **Mitochondrial Membrane Potential (MMP)** | **Phosphoprotein (Tumor Supressor) p53** | **ATPase family AAA domain-containing protein 5 (ATAD5)** |
| **L1** | Inactive | Inactive | Inactive | Inactive | Inactive |
| **L2** | Inactive | Inactive | Inactive | Inactive | Inactive |
| **L3** | Inactive | Inactive | Inactive | Inactive | Inactive |
| **L4** | Inactive | Inactive | Inactive | Inactive | Inactive |
| **L5** | Inactive | Inactive | Inactive | Inactive | Inactive |
| **L8** | Inactive | Inactive | Inactive | Inactive | Inactive |
| **L9** | Inactive | Inactive | Inactive | Inactive | Inactive |
| **L10** | Inactive | Inactive | Inactive | Inactive | Inactive |
| **L14** | Inactive | Inactive | Inactive | Inactive | Inactive |
| **L15** | Inactive | Inactive | Inactive | Inactive | Inactive |
| **L17** | Inactive | Inactive | Inactive | Inactive | Inactive |
| **L24** | Inactive | Inactive | Active | Inactive | Active |
| **L28** | Inactive | Inactive | Active | Active | Active |
| **L30** | Inactive | Inactive | Active | Active | Active |
| **L33** | Inactive | Inactive | Active | Inactive | Active |
| **L35** | Inactive | Inactive | Active | Inactive | Inactive |
| **C** | Inactive | Inactive | Inactive | Inactive | Inactive |
| **Tamoxifen** | Inactive | Inactive | Inactive | Inactive | Inactive |
| **Raloxifen** | Active | Active | Active | Active | Inactive |

**Table S6** Interactions of selected ligands with proteins related to molecular initiating events

| Ligands | Molecular Initiating Events | | | | | | | | | | | | | |
|---|---|---|---|---|---|---|---|---|---|---|---|---|---|---|
| | THRα | THRβ | TTR | RYR | GABAR | NMDAR | AMPAR | KAR | AChE | CAR | PXR | NADHOX | VGSC | NIS |
| **L1** | Inactive | Inactive | Active | Inactive | Inactive | Inactive | Inactive | Inactive | Inactive | Inactive | Inactive | Inactive | Inactive | Inactive |
| **L2** | Inactive | Inactive | Active | Inactive | Inactive | Inactive | Inactive | Inactive | Inactive | Inactive | Active | Inactive | Inactive | Inactive |
| **L3** | Inactive | Inactive | Active | Inactive | Inactive | Inactive | Inactive | Inactive | Inactive | Inactive | Inactive | Inactive | Inactive | Inactive |
| **L4** | Inactive | Inactive | Active | Inactive | Inactive | Inactive | Inactive | Inactive | Inactive | Inactive | Inactive | Inactive | Inactive | Inactive |
| **L5** | Inactive | Inactive | Active | Inactive | Inactive | Inactive | Inactive | Inactive | Inactive | Inactive | Inactive | Inactive | Inactive | Inactive |
| **L8** | Inactive | Inactive | Inactive | Inactive | Inactive | Inactive | Inactive | Inactive | Inactive | Inactive | Inactive | Inactive | Inactive | Inactive |
| **L9** | Inactive | Inactive | Active | Inactive | Inactive | Inactive | Inactive | Inactive | Inactive | Inactive | Inactive | Inactive | Inactive | Inactive |
| **L10** | Inactive | Inactive | Active | Inactive | Inactive | Inactive | Inactive | Inactive | Inactive | Inactive | Inactive | Inactive | Inactive | Inactive |
| **L14** | Inactive | Inactive | Active | Inactive | Inactive | Inactive | Inactive | Inactive | Inactive | Inactive | Inactive | Inactive | Inactive | Inactive |
| **L15** | Inactive | Inactive | Active | Inactive | Inactive | Inactive | Inactive | Inactive | Inactive | Inactive | Inactive | Inactive | Inactive | Inactive |
| **L17** | Inactive | Inactive | Active | Inactive | Active | Inactive | Inactive | Inactive | Inactive | Inactive | Inactive | Inactive | Inactive | Inactive |
| **L24** | Inactive | Inactive | Inactive | Inactive | Inactive | Inactive | Inactive | Inactive | Inactive | Inactive | Inactive | Inactive | Inactive | Inactive |
| **L28** | Inactive | Inactive | Inactive | Inactive | Inactive | Inactive | Inactive | Inactive | Inactive | Inactive | Inactive | Inactive | Inactive | Inactive |
| **L30** | Inactive | Inactive | Inactive | Inactive | Inactive | Inactive | Inactive | Inactive | Inactive | Inactive | Inactive | Inactive | Inactive | Inactive |
| **L33** | Inactive | Inactive | Inactive | Inactive | Inactive | Inactive | Inactive | Inactive | Inactive | Inactive | Inactive | Inactive | Inactive | Inactive |
| **L35** | Inactive | Inactive | Inactive | Inactive | Inactive | Inactive | Inactive | Inactive | Inactive | Inactive | Inactive | Inactive | Inactive | Inactive |

| | | | | | | | | | | | | | | |
|---|---|---|---|---|---|---|---|---|---|---|---|---|---|---|
| **C** | Inactive | Inactive | Inactive | Inactive | Inactive | Inactive | Inactive | Inactive | Inactive | Inactive | Inactive | Inactive | Inactive | Inactive |
| **Tamoxifen** | Inactive | Inactive | Inactive | Inactive | Inactive | Inactive | Inactive | Inactive | Active | Inactive | Inactive | Inactive | Inactive | Inactive |
| **Raloxifen** | Inactive | Inactive | Inactive | Inactive | Inactive | Inactive | Inactive | Inactive | Active | Inactive | Inactive | Inactive | Inactive | Inactive |

Thyroid hormone receptor alpha,THRα; Thyroid hormone receptor beta, THRβ; Transtyretrin, TTR; Ryanodine receptor, RYR; GABA receptor, GABAR; Glutamate N-methyl-D-aspartate receptor, NMDAR; alpha-amino-3-hydroxy-5-methyl-4-isoxazolepropionate receptor, AMPAR; Kainate receptor, KAR; Achetylcholinesterase, AChE; Constitutive androstane receptor, CAR; Pregnane X receptor, PXR; NADH-quinone oxidoreductase, NADHOX; Voltage gated sodium channel, VGSC; Na+/I- symporter, NIS.
